# Primary central nervous system lymphomas: EHA–ESMO Clinical Practice Guideline for diagnosis, treatment and follow‐up

**DOI:** 10.1002/hem3.89

**Published:** 2024-06-04

**Authors:** Andreas J. M. Ferreri, Gerald Illerhaus, Jeanette K. Doorduijn, Dorothee P. Auer, Jacoline E. C. Bromberg, Teresa Calimeri, Kate Cwynarski, Christopher P. Fox, Khê Hoang‐Xuan, Denis Malaise, Maurilio Ponzoni, Elisabeth Schorb, Carole Soussain, Lena Specht, Emanuele Zucca, Christian Buske, Mats Jerkeman, Martin Dreyling, EHA and ESMO Guidelines Committees

**Affiliations:** ^1^ Lymphoma Unit, IRCCS San Raffaele Scientific Institute Milan Italy; ^2^ Università Vita e Salute San Raffaele Milan Italy; ^3^ Department of Hematology Oncology, Stem‐Cell Transplantation and Palliative Care, Klinikum Stuttgart Stuttgart Germany; ^4^ Department of Haematology, Erasmus MC Cancer Institute University Medical Center Rotterdam Rotterdam The Netherlands; ^5^ Mental Health & Clinical Neurosciences Unit, School of Medicine University of Nottingham Nottingham UK; ^6^ NIHR Nottingham Biomedical Research Centre University of Nottingham Nottingham UK; ^7^ Department of Neuro‐Oncology, Erasmus MC Cancer Institute University Medical Center Rotterdam Rotterdam The Netherlands; ^8^ Department of Haematology University College Hospital London UK; ^9^ School of Medicine University of Nottingham Nottingham UK; ^10^ Department of Neurology 2 Mazarin APHP, Groupe Hospitalier Pitié‐Salpêtrière, Sorbonne Université, ICM Paris France; ^11^ Department of Ophthalmology Institut Curie Paris France; ^12^ LITO, INSERM U1288, Institut Curie PSL University Orsay France; ^13^ Pathology Unit, IRCCS San Raffaele Scientific Institute Milan Italy; ^14^ Department of Medicine I, Medical Center, Faculty of Medicine University of Freiburg Freiburg Germany; ^15^ Clinical Hematology Unit, Institut Curie St Cloud France; ^16^ INSERM U932, Institut Curie PSL Research University Paris France; ^17^ Department of Oncology, Rigshospitalet University of Copenhagen Copenhagen Denmark; ^18^ Clinic of Medical Oncology, Oncology Institute of Southern Switzerland, Ente Ospedaliero Cantonale Bellinzona Switzerland; ^19^ Institute of Oncology Research, Faculty of Biomedical Sciences, Università della Svizzera Italiana Bellinzona Switzerland; ^20^ Department of Medical Oncology Bern University Hospital and University of Bern Bern Switzerland; ^21^ Institute of Experimental Cancer Research, Comprehensive Cancer Center Ulm University Hospital of Ulm Ulm Germany; ^22^ Department of Oncology Skåne University Hospital and Lund University Lund Sweden; ^23^ Department of Medicine III LMU University Hospital Munich Munich Germany

## Abstract

- This EHA‐ESMO Clinical Practice Guideline provides key recommendations for managing primary DLBCL of the CNS.
- The guideline covers clinical, imaging and pathological diagnosis, staging and risk assessment, treatment and follow‐up.
- Algorithms for first‐line and salvage treatments are provided.
- The author group encompasses a multidisciplinary group of experts from different institutions and countries in Europe.
- Recommendations are based on available scientific data and the authors' collective expert opinion.

## DEFINITION, INCIDENCE AND EPIDEMIOLOGY

Primary diffuse large B‐cell lymphoma (DLBCL) of the central nervous system (CNS), termed primary CNS lymphoma (PCNSL), is an aggressive neoplasm presenting with disease limited to the CNS. PCNSL was recognised as a distinct entity by the 2017 World Health Organization (WHO) Classification of Tumours of Haematopoietic and Lymphoid Tissues.^1^ In the 2022 edition of the WHO classification,^2^ this neoplasm is classified in the ‘Large B‐cell lymphomas of immune‐privileged sites' group, whereas it is considered a specific entity in the International Consensus Classification of Mature Lymphoid Neoplasms.^3^ This entity is also recognised by the WHO classification of CNS tumours.^4^ PCNSL can arise in both immunocompetent individuals and in those who are immunosuppressed (e.g. individuals living with human immunodeficiency virus and patients receiving immunosuppressive therapies following organ transplant). While no clear predisposing factors have been recognised in immunocompetent individuals, the nature, intensity and duration of immune suppression can influence the risk of PCNSL in those who are immunocompromised.^5^

This European Hematology Association (EHA)–European Society for Medical Oncology (ESMO) Clinical Practice Guideline (CPG) includes recommendations for the management of immunocompetent patients with PCNSL. In this population, PCNSL accounts for 2% of all primary CNS tumours and 4%‐6% of extranodal lymphomas, with an incidence of 0.47/100 000 person‐years.^6^ PCNSL is typically diagnosed in the sixth or seventh decade of life, with a median age at diagnosis of 68 years and a slightly higher frequency in males.^7^ Notably, the recent increase in incidence is limited to patients of >60 years. The incidence of PCNSL in African‐American males of <50 years is more than twofold higher than that in Caucasian males of the same age.^6^ Among elderly patients, however, incidence in Caucasian males is twofold higher than that in African‐American males. Similar patterns, but with a lesser magnitude, are evident among females.^6^

## DIAGNOSIS, PATHOLOGY AND MOLECULAR BIOLOGY

### Clinical presentation

**Figure 1:**
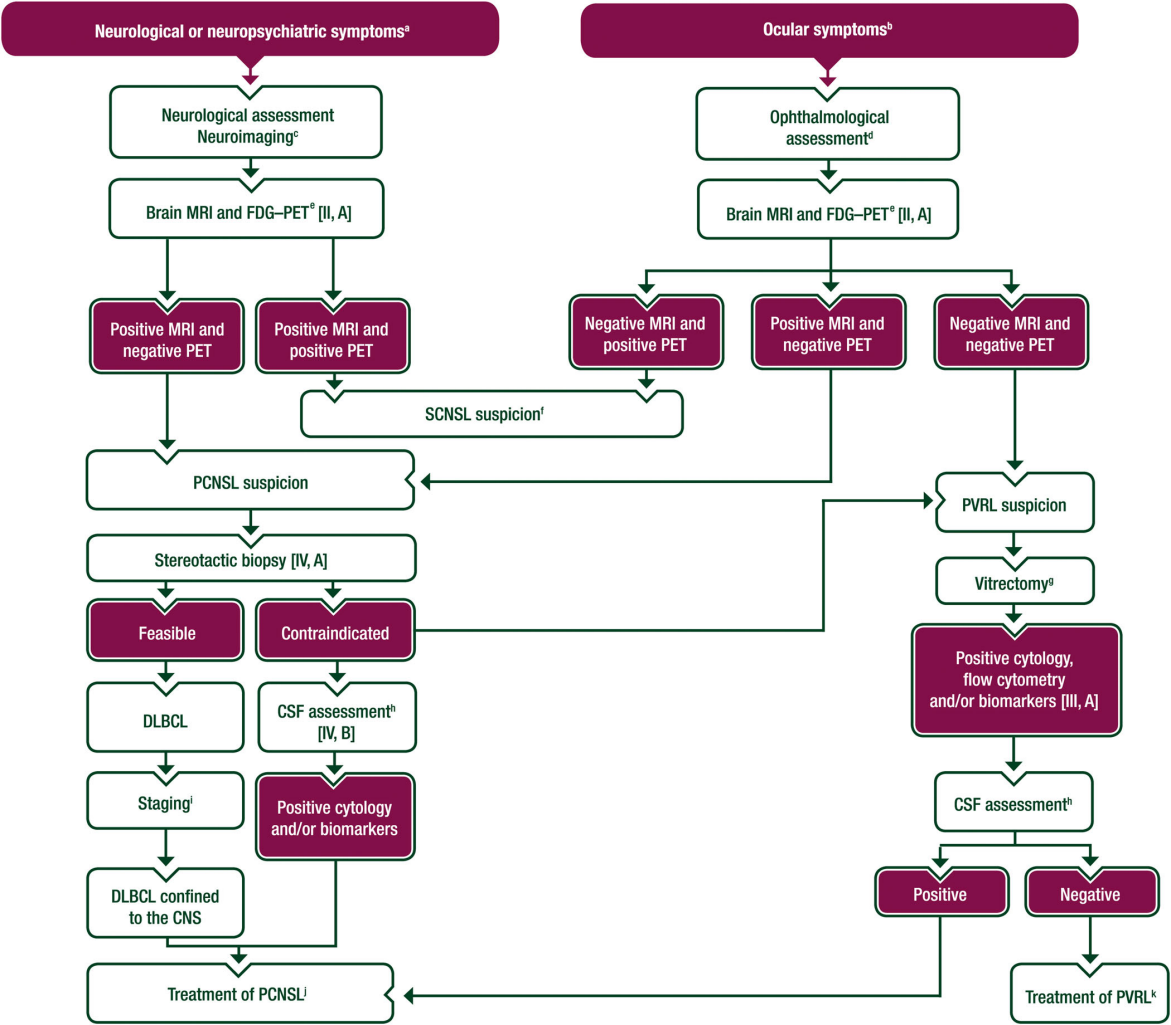
**Diagnostic algorithm for PCNSL in immunocompetent patients**. Purple: general categories or stratification; white: other aspects of management. ADC, apparent diffusion coefficient; CNS, central nervous system; CSF, cerebrospinal fluid; DLBCL, diffuse large B‐cell lymphoma; FDG–PET, [^18^F]2‐fluoro‐2‐deoxy‐d‐glucose–positron emission tomography; IgVH, immunoglobulin heavy chain variable; IL, interleukin; MRI, magnetic resonance imaging; NHL, non‐Hodgkin lymphoma; PCNSL, primary central nervous system lymphoma; PET, positron emission tomography; PVRL, primary vitreoretinal lymphoma; SCNSL, secondary central nervous system lymphoma. ^a^The most common neurological symptoms are focal deficits (70%; hemiparesis, ataxia), neuropsychiatric symptoms or personality changes (43%), high intracranial pressure (33%), seizures (14%), headache, confusion, cognitive dysfunction and lethargy; seizures occur rarely. ^b^The most common ocular symptoms are blurred vision and floaters. ^c^See Supplementary Table S1, available at https://doi.org/10.1016/10.1016/j.annonc.2023.11.010. ^d^See Supplementary Table S5, available at https://doi.org/10.1016/10.1016/j.annonc.2023.11.010. ^e^PCNSL suspicion on MRI is usually based on site of disease and MRI features. The most common sites of disease are frontal lobe and other areas of the brain hemispheres (38%), thalamus or basal ganglia (16%), corpus callosum (14%), periventricular regions (12%), cerebellum (9%), meninges (16%), spinal cord (1%) and cranial and spinal nerves (<1%). MRI features are reported in Supplementary Table S2, available at https://doi.org/10.1016/10.1016/j.annonc.2023.11.010. In particular, lesions are hypointense on T1, isointense to hypointense on T2, reduced ADC, with variable surrounding oedema, homogeneous and often strong enhancement. PET positivity can be indicative of disease outside the CNS. ^f^Consider surgical or endoscopic biopsy of systemic disease and, if a diagnosis of NHL is made, consider treatment for SCNSL. ^g^Suggested exams on vitrectomy samples include conventional cytology, flow cytometry, *MYD88* mutational analysis, level of IL‐6 and IL‐10. Biomarkers can be assessed on anterior chamber samples. ^h^CSF analyses include physical–chemical analysis, conventional cytology examination, flow cytometry, *MYD88 L265P*, IL‐10 level and IgVH clonality in selected cases. ^i^See Supplementary Table S5, available at https://doi.org/10.1016/10.1016/j.annonc.2023.11.010. ^j^See Figure 2. ^k^See Figure 3.

A proposed algorithm for the diagnosis of PCNSL is shown in Figure 1. Patients usually present with a range of neurological or neuropsychiatric symptoms corresponding to the location and extent of the tumour, while systemic symptoms (fever, night sweats and weight loss) are exceptionally rare.^8^ The brain is by far the most common location, with frequent involvement of the corpus callosum, basal ganglia and periventricular areas. Up to 40%‐50% of patients have multifocal disease on standard magnetic resonance imaging (MRI),^7^ resulting in a more complex pattern of symptoms.

The eye is an important but less common site of disease: vitreous fluid and/or the retina are involved in 15%‐20% of patients at presentation, which is asymptomatic in half of the patients while manifesting with blurred vision or floaters in the other half (Figure 1).^7^, ^8^ Ocular involvement is commonly bilateral and can be detected in two different conditions: as the exclusive site of disease or concomitant to other CNS sites, usually brain parenchymal lesions. When disease is limited to the eyes, patients may be initially diagnosed with uveitis, resulting in significant diagnostic delays. If comprehensive staging (see below) excludes the involvement of systemic organs and CNS localisations other than the eye, a diagnosis of primary vitreoretinal lymphoma (PVRL) should be considered. In patients with PVRL, an expert ophthalmologist should exclude differential diagnoses among the ‘masquerade syndromes’, and definitive diagnosis requires cytology examination of vitreous humour. Often, PVRL precedes brain lesions for months or years. When ocular disease is concomitant to other CNS sites, ocular involvement is detected during staging in patients with PCNSL diagnosed on brain biopsy. In both forms, ocular evaluation should be carried out with a slit lamp, fundoscopy and, if required, retinal angiography or tomography.^9^ In patients with a suitable volume of vitreous sample, flow cytometry and PCR assessment of genes encoding immunoglobulin (Ig) heavy chains can be carried out. Although not diagnostic, detection of the *MYD88 L265P* mutation and elevated interleukin‐10 (IL‐10) levels in the vitreous and aqueous humours are indicators of ocular lymphoma.^10^, ^11^

PCNSL can spread through the cerebrospinal fluid (CSF). Concurrent leptomeningeal involvement, which is often asymptomatic, is detected by conventional CSF cytology examination in 16% of patients, while isolated leptomeningeal lymphoma represents <5% of all PCNSLs.^8^ Spinal cord lymphoma is the rarest manifestation of PCNSL and is often associated with a delayed diagnosis and poor prognosis.^6^ Lymphomas arising primarily in the spinal nerves and ganglia, cauda equina and peripheral nerves (‘neurolymphomatosis’) are extremely rare and should be distinguished from neural infiltration of a systemic lymphoma.

### Imaging

Early recognition of imaging features suggestive of PCNSL is essential to expedite definitive tissue diagnosis and avoid corticosteroids before biopsy. The imaging method of choice is contrast‐enhanced MRI including diffusion‐ and perfusion‐weighted scans with volumetric protocols in line with the published recommended minimum and desirable standards of the International PCNSL Collaborative Group (IPCG) (Supplementary Table S1, available at https://doi.org/10.1016/10.1016/j.annonc.2023.11.010).^12^ Patients with contraindications to MRI can be assessed by contrast‐enhanced computed tomography (CT) scan. Typical MRI findings are summarised in Supplementary Table S2, available at https://doi.org/10.1016/10.1016/j.annonc.2023.11.010. The combined use of modern neuroimaging and biomarkers can be useful in distinguishing PCNSL from neoplastic and non‐neoplastic lesions, including neuroinflammatory diseases, brain metastases, non‐necrotic brain tumours, abscesses or tumefactive demyelination.

### Diagnosis

A delay in diagnosis of weeks to months following the onset of symptoms is common in patients with PCNSL.^13^ A critical consideration in the diagnostic pathway is to avoid corticosteroids before biopsy, given their rapid effect on tumour cell viability. Accordingly, if PCNSL is suspected based on imaging and corticosteroids have already been administered, it may be necessary to stop them before stereotactic biopsy of an enhancing lesion. A repeat MRI scan may be required after stopping corticosteroids and before a biopsy.

The gold standard diagnostic method in PCNSL relies on the histopathological examination of specimens obtained by stereotactic biopsy. Surgical resection and/or cytological examination of CSF should not be considered first‐choice diagnostic methods due to the higher morbidity rate conferred by the former and the low diagnostic reliability of the latter.^13^ Histopathological and molecular findings are summarised in Supplementary Table S3, available at https://doi.org/10.1016/10.1016/j.annonc.2023.11.010.

CSF samples should be collected from all patients with suspected or confirmed PCNSL for diagnosis and staging. However, lumbar puncture is not always safe, particularly in patients with concurrent brain masses and/or extensive perilesional oedema. CSF examinations include physical–chemical parameters, conventional cytology and flow cytometry. CSF from patients with PCNSL often has a normal glucose concentration, increased leukocyte count and increased protein concentration. Although CSF examination facilitates diagnosis of PCNSL in <20% of patients, this source of material should be exploited diagnostically. Flow cytometry allows the detection of monotypic B cells, increasing diagnostic sensitivity. Combined assessment of the *MYD88 L265P* mutation, IL‐10 levels and PCR for Ig heavy chain variable (IgVH) rearrangement in the CSF allows efficient discrimination of PCNSL from glial neoplasms and non‐neoplastic disorders of the CNS.^14^ Assessment of these biomarkers in CSF represents a promising tool when biopsy is not possible (e.g. poor clinical condition, brainstem lesions). This approach may also more efficiently identify candidates for stereotactic biopsy among patients with confounding radiological features, i.e. during corticosteroid therapy.

### Molecular biology

PCNSL displays perturbation of pathways related to signalling of B‐cell receptors (BCRs), toll‐like receptors (TLRs) and nuclear factor‐κB (NF‐κB), as well as terminal B‐cell differentiation, deregulation of the cell cycle, immune escape and protection from apoptosis. Among these, the BCR–TLR–NF‐κB axis, mammalian target of rapamycin (mTOR)–protein kinase B (Akt) pathway and programmed cell death protein 1 (PD‐1) have already been targeted therapeutically in PCNSL (see Novel therapies subsection). The biological rationales for potential future therapies are summarised in Supplementary Table S4, available at https://doi.org/10.1016/10.1016/j.annonc.2023.11.010.

### Recommendations

- Contrast‐enhanced cranial MRI is the recommended imaging modality for patients with PCNSL [II, A]. The IPCG protocol based on 3T or 1.5T MRI is recommended [V, A].
- PCNSL diagnosis must be confirmed by histopathological examination of tumour biopsy [III, A].
- Corticosteroid therapy before tissue biopsy should be avoided whenever clinically possible [IV, D]. In case of clinical deterioration, urgent biopsy should be carried out before the start of corticosteroids [IV, A].
- Tissue samples should be collected by stereotactic biopsy in patients with brain lesions [IV, A].
- Tumour resection is not recommended, except in carefully selected patients with rapidly increasing intracranial pressure who may benefit from surgical debulking at the time of tumour biopsy [IV, D].
- Diagnosis is based on morphology and immunohistochemistry [minimum stain panel includes cluster of differentiation (CD)20, CD3, CD10, B‐cell lymphoma (Bcl)‐6, Bcl‐2, multiple myeloma 1 (MUM1) and Ki‐67 antibodies]. Molecular analysis of Ig heavy and light chain loci can be used in selected cases where diagnosis is difficult [V, A].
- When brain biopsy is contraindicated, CSF examination is a valid option. Flow cytometry, *MYD88 L265P* mutation analysis and IL‐10 levels in CSF samples may support a diagnosis of PCNSL [IV, B].
- Suspicion of PVRL should be confirmed by conventional cytology examination of the vitreous humour and, when possible, by flow cytometry. Although not diagnostic, *MYD88 L265P* mutation and IL‐10 levels may be assessed in the vitreous and aqueous humours as indicators of ocular lymphoma [III, A].

## STAGING AND RISK ASSESSMENT

A comprehensive assessment of the extent of lymphoma involvement (see Supplementary Table S5, available at https://doi.org/10.1016/10.1016/j.annonc.2023.11.010) is mandatory to determine both the compartments involved within the CNS and the presence of concomitant systemic disease, as recommended by the IPCG guidelines.^13^ Full neurological and oncohaematological evaluation is crucial before treatment planning. Gadolinium‐enhanced MRI is the most relevant tool to define an extension of disease in the brain and spinal cord. Brain MRI should be repeated after biopsy and ideally within 14 days before starting treatment^12^; this is supported by extremely high proliferative activity, often with >90% of tumour cells expressing the Ki‐67 antigen, which could potentially affect therapeutic response definition.^1^ The involvement of spinal cord parenchyma is rare and specific MRI should be carried out only in patients with symptoms suggestive of spinal cord injury. Meningeal dissemination is often asymptomatic; thus, CSF analysis is advised in every patient with suspected or confirmed PCNSL, unless clinically contraindicated. Physical–chemical features in the CSF (i.e. normal glucose concentration, increased leukocyte count, high protein concentration) are not specific for PCNSL but may suggest meningeal dissemination and blood–CSF barrier disruption. Conventional cytology examination underestimates CSF involvement and should be coupled with flow cytometry to improve diagnostic sensitivity.^15^

Accurate ophthalmological examination should be carried out in every patient with PCNSL. Vitrectomy, however, is not mandatory in patients with histopathological diagnosis of PCNSL carried out on brain biopsies. Assessment of IL‐10 level, *MYD88 L265P* and monoclonal IgVH rearrangement on vitreous and aqueous humours offers diagnostic potential as a conservative procedure to confirm intraocular disease during staging, but its precise role in routine practice remains to be defined.

Assessment for extra‐CNS disease is relevant as patients with PCNSL and secondary CNS lymphoma (SCNSL) exhibit different prognoses and require different treatment protocols.^16^ Conventional staging with [^18^F]2‐fluoro‐2‐deoxy‐d‐glucose–positron emission tomography (FDG–PET), preferably combined with contrast‐enhanced CT scan, can identify systemic disease in 4%‐12% of patients with a presumptive diagnosis of PCNSL.^16^ When FDG–PET is not available, bone marrow biopsy and aspiration and testicular ultrasound (US) are recommended to accompany CT imaging.

To predict outcomes and better stratify patients in clinical trials, two scoring systems have been proposed: the International Extranodal Lymphoma Study Group (IELSG) score^17^ and the Memorial Sloan Kettering Cancer Center prognostic score.^18^ Validation of other proposed scores is pending.

Before starting treatment, bone marrow status and cardiac, liver and renal functions should be assessed (see Supplementary Table S5, available at https://doi.org/10.1016/10.1016/j.annonc.2023.11.010). A battery of cognitive functions and quality of life (QoL) measures has been proposed by the IPCG.^19^ Its use outside clinical trials remains to be defined.

### Recommendations

- Spinal cord imaging should be carried out in symptomatic patients or in case of CSF positivity [V, B].
- Unless lumbar puncture is clinically contraindicated, physical–chemical features of CSF as well as conventional cytology and flow cytometry should be assessed in all patients [IV, B].
- Ophthalmological assessment by slit lamp fundoscopy should be carried out in all patients to exclude intraocular involvement [IV, A]. When available, retinal angiography or tomography is advisable [IV, C].
- All patients should undergo systemic imaging to exclude extra‐CNS disease using FDG–PET, preferably combined with contrast‐enhanced CT scan [V, B].
- If FDG–PET–CT is not feasible, contrast‐enhanced total‐body CT scan, bone marrow aspiration and biopsy and testicular US should be carried out [V, B].

## TREATMENT OF NEWLY DIAGNOSED PCNSL

**Figure 2:**
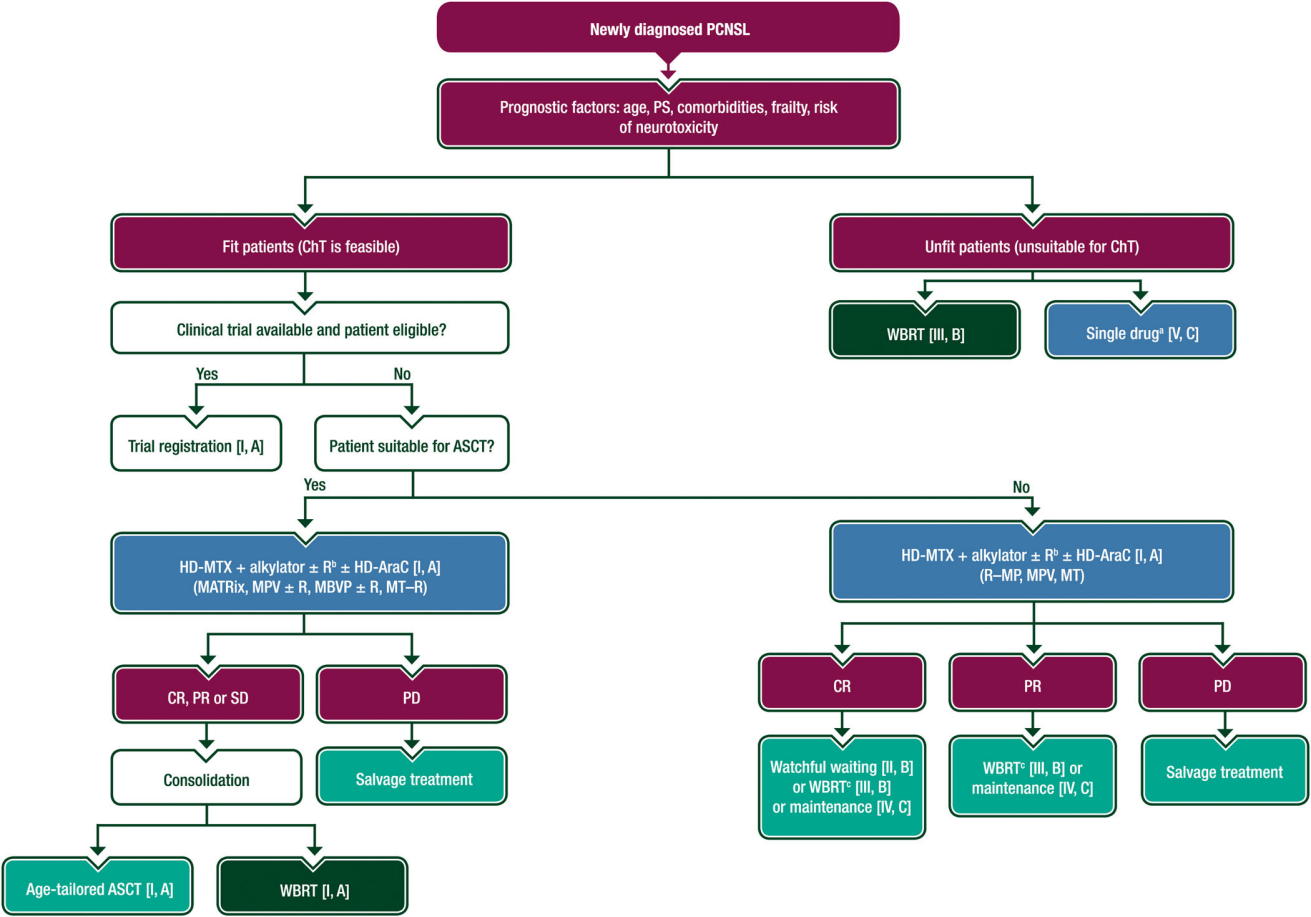
**Treatment algorithm for newly diagnosed PCNSL**. Purple: general categories or stratification; green: RT; blue: systemic anticancer therapy; turquoise: combination of treatments or other systemic treatments; white: other aspects of management. ASCT, autologous stem cell transplantation; ChT, chemotherapy; CR, complete remission; EMA, European Medicines Agency; FDA, Food and Drug Administration; HD‐AraC, high‐dose cytarabine; HD‐MTX, high‐dose methotrexate; MATRix, high‐dose methotrexate–high‐dose cytarabine–rituximab–thiotepa; MBVP, methotrexate–carmustine–teniposide–methylprednisolone; MPV, methotrexate–procarbazine–vincristine; MT, methotrexate–temozolomide; MT–R, methotrexate–temozolomide–rituximab; PCNSL, primary central nervous system lymphoma; PD, progressive disease; PR, partial response; PS, performance status; R, rituximab; R–MP, rituximab–methotrexate–procarbazine; RT, radiotherapy; SD, stable disease; WBRT, whole‐brain radiotherapy. ^a^See text for drug options. ^b^Rituximab is not EMA or FDA approved in this setting; its use in induction combinations remains a matter of debate and the balance between tolerability and efficacy should be discussed with patients and their carers. ^c^Reduced‐dose WBRT.

A proposed algorithm for the treatment of newly diagnosed PCNSL is shown in Figure 2.

### Patient stratification and response assessment

Age is the main prognostic factor in PCNSL. A reliable age cut‐off by which to distinguish young and elderly patients remains to be defined. This has become an increasingly pressing issue in the last decade due to the wider use of high‐dose chemotherapy (HDC) and autologous stem cell transplantation (ASCT), and the increasing incidence of PCNSL with age.^7^, ^20^ The management of older patients with PCNSL remains a clinical challenge, with disappointing survival figures. Comorbidities and neurocognitive dysfunction influence individualised treatment approaches, particularly in patients aged 65‐75 years. Moreover, the use of high‐dose (HD) methotrexate (MTX), the most important component of chemotherapy (ChT) regimens used as first‐line treatment (see Supplementary Table S6, available at https://doi.org/10.1016/10.1016/j.annonc.2023.11.010), requires suitable renal (creatinine clearance >50 ml/min), hepatic and cardiac (left ventricular ejection fraction >45%) functions. Accordingly, stratification between ‘young’ and ‘elderly’ patients should not be made considering exclusively the patient's age but also the ability to tolerate intensified treatments, informed by performance status (PS), organ function, comorbidities and frailty. Importantly, given the rarity of PCNSL and the complexity of its management, the overall evaluation and treatment of every patient should be carried out in specialist centres by an experienced multidisciplinary team, which should involve neurosurgeons, neuroradiologists, haematopathologists, haematologists, oncologists, radiation oncologists and ophthalmologists with specialist knowledge of PCNSL. Moreover, enrolment in a prospective clinical trial should always be prioritised. Most of the following recommendations concern treatment in routine practice.

Response to treatment should follow IPCG criteria^13^: gadolinium‐enhanced MRI of the brain should be carried out every two courses during induction ChT and 2 months after consolidation, and compared with baseline MRI, with the addition of ocular and CSF exams if involved at baseline.

### First‐line treatment for fit patients suitable for HDC–ASCT

#### Induction treatment

Due to superior efficacy, intensive systemic ChT protocols have replaced the historical therapeutic standard of whole‐brain radiotherapy (WBRT) as initial treatment of patients with PCNSL. Empirically adopted from systemic lymphoma protocols, combination ChT regimens such as cyclophosphamide–doxorubicin–vincristine–prednisone (CHOP) have proven ineffective due to their insufficient ability to cross the blood–brain barrier.^21^ HD‐MTX is widely established as a key component of current PCNSL remission induction protocols. HD‐MTX doses of at least 3 g/m^2^ with a rapid infusion time of 2‐4 h are recommended to achieve sufficient drug levels in the CNS; some experts advise preceding MTX infusion with a fast bolus (MTX 500 mg/m^2^ infused over 15 min). The central role of HD‐MTX has been confirmed in several prospective non‐randomised clinical studies either as monotherapy^22^ or as polychemotherapy (polyChT).^23^, ^24^ Recently, the approach to upfront induction therapy has evolved to be increasingly intensive, translating into improved efficacy and higher chances of long‐term remission or cure.

HD cytarabine (AraC) 2 g/m^2^ every 12 h for 2 days has also been shown to be an important component of the treatment of PCNSL. This was first demonstrated in the randomised phase II IELSG20 trial.^24^ Compared with HD‐MTX monotherapy (3.5 g/m^2^), the HD‐MTX–HD‐AraC combination was associated with a significantly increased overall response rate (ORR) (40% versus 69%, respectively) and progression‐free survival (PFS) (3‐year PFS 21% versus 38%), as well as a trend in favour of the combination in overall survival (OS) (3‐year OS 32% versus 46%).

Based on these results, the international randomised phase II IELSG32 trial compared three different induction protocols: HD‐MTX–HD‐AraC (standard), HD‐MTX–HD‐AraC–rituximab and HD‐MTX–HD‐AraC–rituximab–thiotepa (MATRix regimen) followed by a second randomisation comparing consolidation with HDC–ASCT versus WBRT.^25^ Two hundred and twenty‐seven patients were registered across 53 centres in five European countries. The MATRix regimen achieved significantly better response, PFS and OS rates.^25^ An updated analysis at a median follow‐up of 88 months confirmed the benefit of MATRix,^26^ with significantly improved 7‐year PFS (52% versus 29% versus 20%) and 7‐year OS (56% versus 37% versus 26%) rates compared with HD‐MTX–HD‐AraC–rituximab and HD‐MTX–HD‐AraC, respectively. Patients who received the MATRix regimen followed by consolidation (either WBRT or HDC–ASCT) achieved a 7‐year OS rate of 70%. Based on these data, MATRix is one of the recommended induction regimens (see Figure 2 and Supplementary Table S6, available at https://doi.org/10.1016/10.1016/j.annonc.2023.11.010), irrespective of the planned consolidation modality, for eligible patients aged ≤65 years with an Eastern Cooperative Oncology Group (ECOG) PS 0‐3 or aged ≤70 years with an ECOG PS ≤2. Of note, a real‐world study involving 156 consecutive patients treated at 13 centres across the UK, Italy and Germany showed that, when used in everyday practice, MATRix is associated with efficacy and tolerability comparable to those reported in the IELSG32 trial, with a 2‐year OS rate of 64% and a 6% treatment‐related mortality (TRM) rate.^27^ Notably, one‐third of treated patients were aged >70 years, had ECOG PS ≥3 or had significant comorbidities, findings often associated with higher toxicity and lower efficacy. Of note, 62% of the study population received all four planned cycles, but three‐quarters of them required dose reductions.

Other regimens in use in Europe are HD‐MTX–carmustine–teniposide–methylprednisolone (MBVP) and rituximab–MBVP (R–MBVP) followed by HD‐AraC. This has been used in the HOVON 105‐ALLG NHL 24 randomised phase III trial, which compared two cycles of MBVP ChT with two courses of R–MBVP, followed by one course of HD‐AraC, and, in patients <60 years, by WBRT. The treatment resulted in a 1‐year PFS rate of 58% and a 3‐year OS rate of 61%, without significant differences between the two induction arms.^28^ In the PRECIS trial, designed to compare two different consolidation strategies (HDC–ASCT versus WBRT), patients received two courses of rituximab–HD‐MTX–carmustine–etoposide–prednisone followed by two doses of HD‐AraC–rituximab as induction.^29^ Updated results showed an 8‐year OS rate of 69% after HDC–ASCT and 65% after WBRT.^30^ Induction therapies without HD‐AraC tested in large prospective trials may also be considered, such as rituximab–MTX–temozolomide (R–MT)^31^ or rituximab–MTX–procarbazine–vincristine (R–MPV)^32^ (see Supplementary Table S6, available at https://doi.org/10.1016/10.1016/j.annonc.2023.11.010).

The role of rituximab was questioned by the HOVON 105‐ALLG NHL 24 trial.^28^ There was no event‐free survival (EFS) advantage with R–MBVP versus the MBVP arm and while PFS was numerically improved with R–MBVP, the increase was not statistically significant. An unplanned analysis demonstrated EFS and PFS benefits with R–MBVP versus MBVP in patients aged ≤60 years, whereas patients aged >60 years seemed to have reduced EFS and PFS with R–MBVP versus MBVP. Conversely, recent results from the IELSG32 trial, published after a median follow‐up of 88 months, demonstrated that addition of rituximab to MTX–AraC was associated with significantly improved PFS and OS,^26^ and a meta‐analysis of study‐level data from the two randomised trials that evaluated the role of rituximab as part of induction therapy (IELSG32 and HOVON 105‐ALLG NHL 24) confirmed a PFS benefit, but with low certainty.^33^

Due to the pharmacokinetic limitations of intravenously (i.v.) delivered drugs in achieving therapeutic concentrations in the CSF and intraocular humour, some experts have proposed intrathecal and/or intravitreal ChT as part of initial treatment.^34^ Evidence regarding the role of these therapies is fragile because it is based on non‐randomised prospective and retrospective studies, with conflicting results and sometimes substantial toxicity. Thus, intrathecal ChT is not routinely recommended if appropriate systemic ChT can be applied; however, it could be considered in the case of isolated meningeal disease persisting after induction polyChT. Intravitreal ChT is used as upfront treatment in selected patients with PVRL (see below), and in cases of intraocular disease with insufficient response to HD‐MTX‐based protocols.

#### Consolidation options

Despite high initial response rates to HD‐MTX‐based therapy, relapses often occur. To eliminate residual disease and reduce relapse risk, consolidation therapy should be carried out. HDC–ASCT and WBRT are the available consolidation strategies.^29^, ^35^

##### HDC–ASCT

HDC–ASCT is the most intensive consolidation therapy and can lead to promising long‐term results in suitable patients. The rationale comprises the administration of maximally dosed blood–brain barrier‐penetrating cytostatics to overcome drug resistance and reach therapeutic drug concentrations in the CNS. Conditioning protocols commonly administered in the treatment of systemic lymphoma [i.e. carmustine bis‐chloroethylnitrosourea–etoposide–AraC–melphalan (BEAM regimen)] have yielded disappointing results in patients with PCNSL,^36^ whereas thiotepa‐containing protocols (see Supplementary Table S7, available at https://doi.org/10.1016/10.1016/j.annonc.2023.11.010) have shown good efficacy.^37^, ^38^ In a multicentre trial involving 79 patients aged ≤65 years with PCNSL,^39^ four courses of rituximab–HD‐MTX followed by rituximab–HD‐AraC–thiotepa and consolidating carmustine–thiotepa‐conditioned ASCT have been associated with 5‐year PFS and OS rates of 65% and 79%, respectively. A prospective single‐centre study of 32 patients aged <65 years treated with R–MPV induction and HDC–ASCT conditioned with thiotepa–busulfan–cyclophosphamide (TBC) reported 2‐year PFS and OS rates of 79% and 81%, respectively, and a TRM rate of 10%.^40^

The effect of consolidation with either HDC–ASCT or WBRT in patients with newly diagnosed PCNSL has been established in two randomised phase II trials: the IELSG32^35^ and PRECIS^29^ studies. The conditioning regimens were carmustine–thiotepa in IELSG32 and TBC in PRECIS (see Supplementary Table S7, available at https://doi.org/10.1016/10.1016/j.annonc.2023.11.010). In IELSG32, similar results were observed in both treatment arms in terms of response and PFS (per protocol 2‐year PFS rate 75% with HDC–ASCT versus 76% with WBRT). In PRECIS, a significant improvement in PFS was observed with HDC–ASCT; per protocol, the 2‐year PFS rate was 86% versus 67% with WBRT. Both trials reported a decline in neurocognitive function in a substantial proportion of patients after WBRT but not after HDC–ASCT. As expected, grade ≥3 febrile neutropaenia and mucositis were common after HDC–ASCT, particularly after TBC conditioning, with a TRM rate of 11%.^29^

HDC–ASCT seems to be a safe and effective therapeutic option for selected elderly patients. A retrospective multicentre study^41^ and two prospective trials (MARiTA and MARTA) investigated HDC–ASCT in patients aged >65 years with PCNSL. In a pilot study, treatment with rituximab–HD‐MTX–HD‐AraC followed by busulfan–thiotepa‐conditioned ASCT was associated with 2‐year PFS and OS rates of >90%, without cases of lethal toxicity.^42^ Haematological toxicity was frequent, but rates of infective complications were similar to those reported after conventional ChT protocols.^43^ Initial results of the MARTA trial showed that patients aged >65 years treated with two courses of rituximab–MTX–AraC and consolidation with rituximab–busulfan–thiotepa‐conditioned ASCT achieved a 1‐year PFS rate of 59%.^44^

Several prospective trials have demonstrated that HDC–ASCT is associated with very good long‐term outcomes in younger and fit patients, and that WBRT can often be avoided. Accordingly, discussion with selected patients about the pros and cons of consolidation HDC–ASCT or WBRT is recommended.

##### Radiotherapy

The long‐term adverse cognitive effects of irradiating the whole brain, particularly among elderly patients,^29^, ^33^, ^45^, ^46^ have led to most patients being consolidated with HDC–ASCT. Nevertheless, WBRT remains a valid alternative for fit patients with insufficient autologous stem cell harvest and for patients who refuse HDC–ASCT. A few patients with residual disease after HDC–ASCT are also candidates for WBRT.

The role of consolidation WBRT has been addressed, with conflicting results, in two randomised trials using (immuno)ChT alone as comparator. Patients enrolled in the G‐PCNSL‐SG‐1 trial were treated with HD‐MTX ± HD‐ifosfamide and, according to response to ChT, patients in complete remission (CR) were randomly allocated to receive WBRT (45 Gy/30 fractions) or observation, whereas all other patients were randomised to WBRT or HD‐AraC monotherapy.^47^ WBRT was associated with improved PFS but no benefit in OS. Unfortunately, these results may have been influenced by methodological limitations, such as major protocol violations in one‐third of enrolled patients.^47^ Initial results from the randomised RTOG1114 trial demonstrated a significant improvement in PFS with the addition of reduced‐dose WBRT to rituximab–HD‐MTX‐based ChT compared with the same ChT alone, with median PFS not reached at 55 months for patients treated with chemoradiotherapy (CRT) and 25 months for patients treated with ChT alone.^31^

PCNSL is multifocal in **∼**40% of cases based on MRI at diagnosis, but conventional MRI is known to underestimate the extent of disease.^48^ Focal radiotherapy (RT) results in increased relapses in areas outside the irradiated volume^49^; thus, the whole brain should be irradiated.^50^, ^51^ Details on RT technique are provided in Supplementary Table S8, available at https://doi.org/10.1016/10.1016/j.annonc.2023.11.010. No randomised trials have compared different WBRT doses, but a dose tailored to the response after induction ChT is recommended.^32^, ^45^ There is an increasing interest in the use of stereotactic RT, radiosurgery and hippocampal sparing in PCNSL.^52^ These options, however, should be used only within clinical trials as prospective evidence and safety data are lacking.

##### Non‐myeloablative ChT

De‐escalation of ASCT, using non‐myeloablative ChT, has been hypothesised as a suitable strategy to improve feasibility and to extend the indication to older patients. This strategy was investigated in a single‐arm phase II study^53^ that preceded two important randomised trials. In the randomised ALLIANCE 51101 study, consolidation with non‐myeloablative HD‐AraC–etoposide was compared with carmustine–thiotepa‐conditioned ASCT in 108 patients aged 18‐75 years with PCNSL.^54^ Nonmyeloablative ChT was associated with poorer 2‐year PFS rates (51% versus 73% with ASCT; *P* = 0.02) and had a similar toxicity profile.^54^ Initial results of an international randomised phase III trial comparing rituximab–dexamethasone–etoposide–carboplatin (R–DeVIC regimen) with carmustine–thiotepa‐conditioned ASCT after induction therapy with four courses of the MATRix regimen (MATRix/IELSG43 trial; NCT02531841) were recently reported.^55^ PFS and OS were significantly improved in the ASCT arm, with 3‐year PFS rates of 79% after HDC–ASCT and 53% after R–DeVIC, and 3‐year OS rates of 86% and 71%, respectively.^55^ The evaluation of neurocognitive functions showed no difference between arms. Long‐term results from these randomised studies will define the value of non‐myeloablative ChT as part of upfront treatment of PCNSL; in the meantime, this remains an experimental approach.

A single‐arm phase II trial by the Nordic Lymphoma Group showed that temozolomide maintenance after HD‐MTX‐based ChT could provide survival benefit in patients aged >65 years with PCNSL.^56^ Conversely, recent results of the randomised phase III JCOG1114C trial suggested that addition of temozolomide as radiomimetic and maintenance does not improve outcomes.^57^ Patients aged 18‐70 years with PCNSL were treated with HD‐MTX monotherapy and randomly allocated to receive consolidation with WBRT alone (control arm) or consolidation with WBRT plus temozolomide followed by 2‐year maintenance with temozolomide (experimental arm), with 2‐year OS rates of 87% and 71%, respectively. The role of temozolomide maintenance in patients treated with more intensified induction (immuno)ChT remains to be defined.

### First‐line treatment for patients unsuitable for HDC–ASCT

Unsuitability for HDC–ASCT in patients with PCNSL is mostly related to advanced age, poor PS, treatment‐related toxicity or comorbidities. Premorbid PS can be used to determine treatment intensity, but this parameter frequently changes during treatment, requiring reassessment after the first ChT courses to redefine therapeutic goals. Treatment benefit is limited in elderly patients because they are often ineligible for HDC–ASCT and at high risk of neurotoxicity after WBRT.^7^

#### Induction ChT

Standard induction treatment for fit, elderly patients is HD‐MTX‐based ChT; however, choice of treatment regimen and delivery of adequate dose intensity are therapeutic challenges. Data from retrospective studies suggest that MTX ≥3 g/m^2^ is well tolerated in most elderly patients, including those aged >80 years,^58^ and that dose intensity reductions appear to be associated with inferior outcomes.^59^ Reducing the dose of MTX is not justified in elderly patients with preserved renal function [estimated glomerular filtration rate (eGFR) ≥50 ml/min] but should be considered in patients with eGFR <50 ml/min.^60^ Combination of HD‐MTX with other cytostatic agents appears to provide some benefit when feasible, although there is no evidence from prospective clinical trials comparing polyChT versus single‐agent ChT in this age group.^61^ Oral alkylating agents, such as procarbazine, lomustine–temozolomide and HD‐AraC–rituximab, have been combined with HD‐MTX in elderly patients.^29^, ^62^, ^63^, ^64^ Although commonly used in several combinations, the indication for rituximab remains debated in elderly patients.^28^, ^33^ Regimens assessed in prospective trials are recommended in routine practice.^31^, ^42^, ^43^, ^65^ In a multicentre randomised phase II trial, 98 patients aged >60 years with PCNSL received HD‐MTX–procarbazine–vincristine–AraC (MPV–A regimen) or HD‐MTX–temozolomide (MT regimen).^31^ ORR was 82% with MPV–A and 71% with MT, with a 2‐year PFS rate of 30% for both arms. Tolerability and toxicity were similar for both regimens. In another prospective, multicentre trial (PRIMAIN), rituximab–procarbazine–HD‐MTX (R–MP regimen) was evaluated in 38 patients aged >65 years and reported 2‐year PFS and OS rates of 37% and 47%, respectively, with a TRM rate of 8%.^43^

#### Consolidation options

Consolidation strategies are relevant to patients unsuitable for HDC–ASCT, aiming to maximise duration of response and survival outcomes while preserving QoL. Randomised trials of consolidation therapies in elderly patients are not available; all consolidation options are supported by single‐arm phase II trials.

Consolidation with WBRT at conventional doses (40 Gy ± boost) has been increasingly abandoned in elderly patients with PCNSL due to the high risk of severe neurotoxicity,^66^ whereas ‘watchful waiting’ is suggested only in patients in CR after well‐established induction immunoChT. Two large retrospective studies have found that WBRT consolidation was adopted in very few patients aged >60 years (2% and 8%, respectively), reflecting an evolution of practice favouring ChT‐based consolidation or maintenance in elderly patients.^7^, ^59^ An important question remains regarding the effect of reduced‐dose WBRT in older patients, in terms of efficacy and neurotoxicity. Preliminary data from single‐arm studies suggest that a WBRT dose of 23.4 Gy for patients in first CR is associated with encouraging survival rates, without evidence of significant cognitive decline.^32^ The number of patients aged >65 years included in these trials, however, was insufficient to draw definitive conclusions. Initial results of the RTOG1114 trial support the use of reduced‐dose WBRT (23.4 Gy/13 fractions), although results from further neuropsychological assessments are pending.^31^ The optimal dose of WBRT to balance efficacy with neurotoxicity is not yet known; there are currently no randomised trials comparing different doses of WBRT.

As discussed earlier, non‐myeloablative ChT is an interesting alternative to HDC–ASCT and WBRT as consolidation, especially in elderly patients.^53^ It should be noted, however, that studies evaluating this strategy in patients aged >70 years are not available and high toxicity rates have been reported in routine practice.^67^

#### Maintenance treatment

Maintenance treatment is feasible and associated with encouraging results in elderly patients.^68^ The phase III BLOCAGE trial (NCT02313389), evaluating the role of maintenance immunoChT (R–MT regimen) versus observation in patients with CR after HD‐MTX‐based induction, is ongoing. Lenalidomide has shown promising results as single‐agent maintenance after induction ChT in a small cohort of elderly patients with PCNSL.^69^ The FIORELLA trial (NCT03495960) will pioneer a randomisation of procarbazine versus lenalidomide as maintenance treatment after R–MP induction.

### First‐line treatment for unfit patients unsuitable for ChT

Less than 15% of patients with PCNSL are considered unfit for HD‐MTX‐containing therapy based on advanced age, frailty or comorbidities. These patients have a very poor prognosis and data to guide treatment decisions in this setting are scarce. Life expectancy, PS and desired QoL should be discussed with patients and their carers. Importantly, best supportive care should be preferred in selected frail patients when symptomatic and PS benefit is not considered achievable. Suitable treatment options include corticosteroids, oral alkylating agents (temozolomide, carmustine, procarbazine) with or without rituximab and WBRT.^70^ When WBRT is used as palliative treatment, a dose of 30‐36 Gy/10 or 15 fractions is suggested.^51^ Novel active drugs such as Bruton tyrosine kinase (BTK) inhibitors and immunomodulators may represent off‐label alternatives for these patients.

### Recommendations

- Enrolment in suitable prospective clinical trials should be offered to every patient with PCNSL [I, A].
- When a prospective trial is not available, induction ChT including HD‐MTX is recommended at a minimum dose of 3 g/m^2^ delivered in a 3‐h infusion [I, A].
- Combinations of HD‐MTX with other cytotoxic agents that cross the blood–brain barrier and have been tested in prospective (preferably randomised) trials are recommended (e.g. MATRix, R–MBVP, rituximab–HD‐MTX–carmustine–etoposide–prednisone, R–MPV, R–MT) [I, A].
- The benefit of adding rituximab [not European Medicines Agency (EMA) approved, not Food and Drug Administration (FDA) approved] to induction HD‐MTX‐based polyChT remains unclear. The balance between tolerability and efficacy should be discussed with patients and their carers [II, B].
- Judicious reduction of MTX dose according to renal function and comorbidities is recommended [III, A].
- Intrathecal ChT is not recommended in routine practice, except for patients with CSF dissemination who are unable to receive ChT including MTX at ≥3 g/m^2^ or for patients with persistence of CSF or meningeal disease at the end of first‐line treatment [III, D].
- Intravitreal ChT is not recommended in routine practice, except for patients with persistent intraocular lymphoma at the end of first‐line treatment [III, D].
- HDC–ASCT is recommended as consolidation in fit patients with responsive or stable disease after suitable induction ChT [I, A].
- Fitness for HDC–ASCT should be evaluated dynamically during treatment, especially in older patients who may gain or lose ‘HDC–ASCT fitness’ during induction ChT [III, B].
- Thiotepa‐based ASCT conditioning regimens should be used. The dose of thiotepa combined with either busulfan or carmustine should be based on established protocols and informed by patient fitness and comorbidities [III, A].
- Consolidation WBRT at a dose of 36‐40 Gy/20 fractions is recommended in young patients who are not suitable candidates for ASCT. Safety profiles (haematological and cognitive toxicities) should be considered for individual therapeutic choice [I, A].
- Consolidation WBRT at a dose of 36‐40 Gy/20‐22 fractions should be avoided or deferred in elderly patients because of the high risk of disabling neurocognitive impairment [I, D].
- Reduced‐dose WBRT (23.4 Gy) is an option for patients with responsive disease after suitable induction ChT, but the longer‐term effects on cognitive function remain to be defined, especially in elderly patients [III, B].
- Watchful waiting can be considered in elderly patients in CR after induction with an established drug combination [II, B]. Maintenance with oral drugs, such as alkylating agents or immunomodulators such as lenalidomide (not EMA approved, not FDA approved) can be considered on an individual basis [IV, C].
- There is no established standard of care for patients unfit for HD‐MTX‐based ChT. Valid (but incompletely investigated) palliative options include upfront WBRT [III, B], corticosteroids, oral alkylating agents with or without rituximab (not EMA approved, not FDA approved), BTK inhibitors and immunomodulators [V, C].
- Assessment of response to treatment should follow modalities and timing proposed by the IPCG criteria [III, B].

## TREATMENT OF PVRL

**Figure 3:**
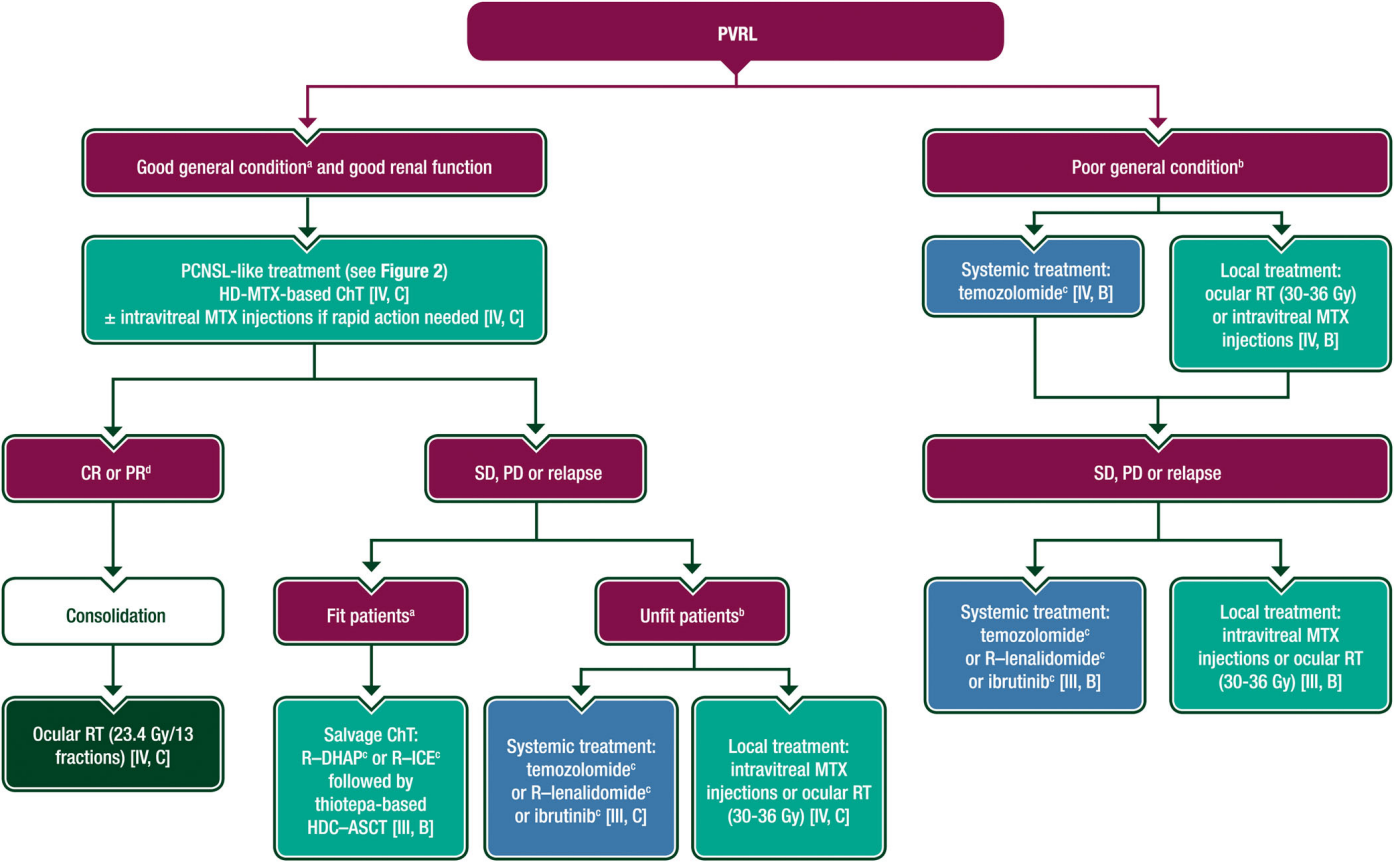
**Treatment algorithm for PVRL**. Purple: general categories or stratification; green: RT; blue: systemic anticancer therapy; turquoise: combination of treatments or other systemic treatments; white: other aspects of management. ChT, chemotherapy; CR, complete remission; EMA, European Medicines Agency; FDA, Food and Drug Administration; HDC–ASCT, high‐dose chemotherapy and autologous stem cell transplantation; HD‐MTX, high‐dose methotrexate; IL‐10, interleukin‐10; IPCG, International PCNSL Collaborative Group; MTX, methotrexate; PCNSL, primary central nervous system lymphoma; PD, progressive disease; PR, partial response; PVRL, primary vitreoretinal lymphoma; R, rituximab; R–DHAP, rituximab–dexamethasone–cytarabine–cisplatin; R–ICE, rituximab–ifosfamide–carboplatin–etoposide; RT, radiotherapy; SD, stable disease. ^a^Patients with no major comorbidities and good clinical condition. ^b^Patients with relevant comorbidities or poor clinical condition at the discretion of the clinician. ^c^Not EMA approved, not FDA approved. ^d^PR is defined according to the IPCG criteria^14^ but with an undetectable level of IL‐10 in aqueous humour.

The therapeutic challenges in PVRL are twofold: to limit visual consequences and to prevent CNS dissemination. Fifty‐six percent to 90% of patients with PVRL develop CNS dissemination within 30 months; this is the main cause of death in patients with PVRL.^63^ Median survival appears to be longer when the patient is treated at the time of PVRL diagnosis rather than at CNS relapse.^71^ Data on heterogeneous treatments are mainly retrospective, with evident bias related to the involved medical specialists (i.e. haematologists or ophthalmologists). Thus, debate persists on the best treatment for PVRL, both at presentation and for relapsed or refractory (r/r) disease. Treatment options include local therapies (intravitreal drug injection or ocular RT), systemic (immuno)ChT or both. A proposed algorithm for the treatment of PVRL is shown in Figure 3.

Antitumour drugs (usually MTX or rituximab) can be injected in the vitreous under local anaesthesia, with varied duration and number of injections.^9^ This approach is used in first‐line treatment or at relapse, either alone (usually in unilateral disease) in patients not eligible for systemic ChT,^66^ or in addition to systemic treatment when rapid antitumour activity is needed. External beam ocular RT is rarely used alone as first‐line treatment in patients with bilateral ocular involvement or as salvage treatment for elderly patients. The recommended technique to irradiate the eyes is summarised in Supplementary Table S8, available at https://doi.org/10.1016/10.1016/j.annonc.2023.11.010. Overall, local treatments seem to be effective in controlling intraocular disease, but they fail to prevent CNS relapses and ocular relapses remain frequent.^9^ Encouraging results were recently reported in patients treated with a combination of bilateral ocular RT and HD‐MTX‐based ChT.^9^, ^72^ However, it is not yet clear whether CNS‐directed polyChT should be given upfront to prevent CNS dissemination.^63^ Systemic treatments for PVRL remain empirical. HD‐MTX‐based polyChT has been frequently used either alone or combined with a local therapy to treat PVRL at presentation^9^; however, control of intraocular disease is poor.^73^ In a recent retrospective study of 59 patients with PVRL receiving HD‐MTX‐based ChT, median survival without brain relapse was prolonged (73 months) and none of the eight patients who received an additional local treatment experienced intraocular relapse after a median follow‐up of 5 years.^73^

Intensive thiotepa‐based ChT followed by ASCT is feasible and effective in fit patients with r/r PVRL.^9^, ^74^ Temozolomide and targeted therapies, such as lenalidomide (alone or in combination with rituximab) or ibrutinib, have demonstrated some activity in r/r PVRL.^75^

### Recommendations

- Fit patients with PVRL should be treated with HD‐MTX‐based induction ChT, using the same combinations proposed for other patients with PCNSL [IV, C]. Intravitreal MTX injections can be added if a rapid regression of intraocular disease is needed [IV, C].
- Patients with a CR or partial response to HD‐MTX‐based induction ChT can be eligible for consolidation with low‐dose bilateral ocular RT [IV, C]. The risks and benefits of consolidation with ASCT can be discussed with selected patients [V, C].
- Therapeutic response should be assessed by slit lamp and fundus examination during and at the end of induction as well as after consolidation; however, precise response definition based on clinical evaluation is difficult. If available, assessment of *MYD88 L265P* and IL‐10 levels in aqueous humour may be useful to monitor intraocular response. Angiography and optical coherence tomography could improve response definition [V, C].
- Oral alkylating agents [i.e. temozolomide (not EMA approved, not FDA approved)] and local therapies (ocular RT or intravitreal MTX injection) are acceptable options as first‐line treatment for unfit patients or patients with contraindications to ChT [IV, B].
- Fit patients with r/r PVRL should be treated with second‐line ChT and consolidative HDC–ASCT [III, B].
- Local treatments, as well as ibrutinib, lenalidomide or temozolomide, are alternative options for patients with r/r PRVL in poor general condition [III, B; not EMA approved, not FDA approved].

## TREATMENT OF R/R PCNSL

Despite therapeutic progress, 16%‐26% of patients aged ≤70 years with PCNSL are primary refractory to HD‐MTX‐based ChT,^7^, ^35^ and a further 25% experience relapse after initial response.^76^ Relapse rates are remarkably higher among older patients. Relapses occur predominantly in the CNS, often in sites distant from the primary lesion.^77^ Most relapses are associated with rapid disease progression and corresponding neurological symptoms; only 20% of relapses are diagnosed on surveillance MRI.^78^, ^79^ The prognosis of patients with r/r PCNSL is very poor, and benefits from salvage therapies are often marginal. Notably, patients who experience relapse after the first 3 years of follow‐up demonstrate a significantly better 2‐year survival rate after relapse (70%) than patients with refractory disease (11%) and those who experience a relapse during the second or third years of follow‐up (12%).^26^

**Figure 4:**
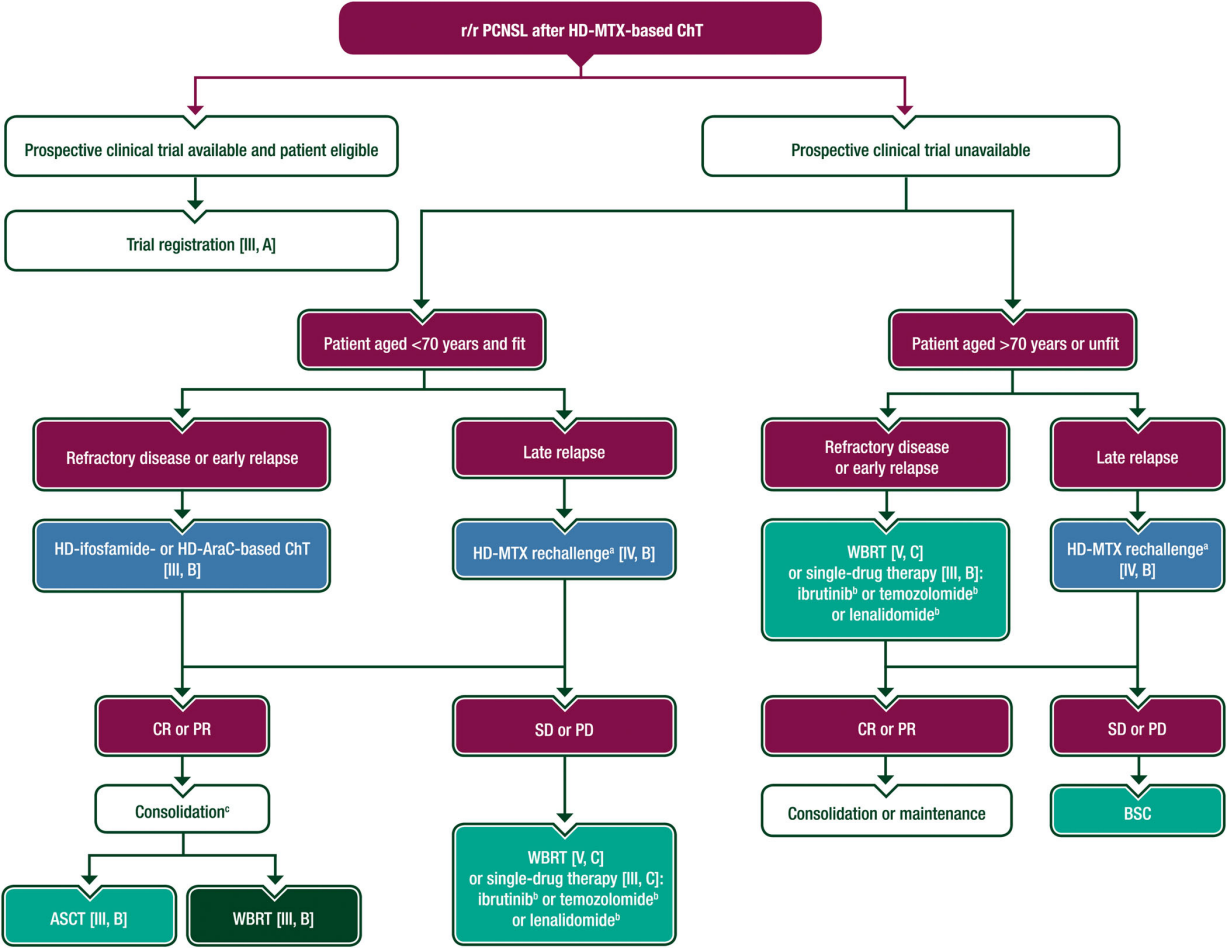
**Treatment algorithm for r/r PCNSL**. Purple: general categories or stratification; green: RT; blue: systemic anticancer therapy; turquoise: combination of treatments or other systemic treatments; white: other aspects of management. ASCT, autologous stem cell transplantation; BSC, best supportive care; ChT, chemotherapy; CR, complete remission; EMA, European Medicines Agency; FDA, Food and Drug Administration; HD, high dose; HD‐AraC, high‐dose cytarabine; HD‐MTX, high‐dose methotrexate; PCNSL, primary central nervous system lymphoma; PD, progressive disease; PR, partial response; r/r, relapsed or refractory; RT, radiotherapy; SD, stable disease; WBRT, whole‐brain radiotherapy. ^a^Drugs and doses vary according to age, comorbidity and frailty. ^b^Not EMA approved, not FDA approved. ^c^Choice of consolidation therapy in fit patients aged <70 years should consider the consolidation strategy used in first‐line treatment; a different consolidation is preferred (i.e. salvage with WBRT if ASCT was used in first‐line treatment and vice versa). A second ASCT may be an option for selected patients, especially those with long‐lasting remission after the first ASCT.

A proposed algorithm for the treatment of r/r PCNSL is shown in Figure 4. HD‐AraC‐ or HD‐ifosfamide‐based ChT followed by consolidative HDC–ASCT is an option for fit patients.^79^, ^80^ HD‐MTX rechallenge can result in a second durable remission in patients who experience long‐lasting regression after a previous HD‐MTX‐based combination.^81^ Patients with contraindications to ChT can be treated with salvage WBRT, with a reported median OS of 11 months.^82^ Less than 5% of relapses occur outside the CNS^7^; these patients may achieve remission with rituximab–cyclophosphamide–doxorubicin–vincristine–prednisone (R–CHOP) immunoChT.^8^

### Novel therapies

Some of the abnormalities that characterise the genomic profile of PCNSL (see Supplementary Table S4, available at https://doi.org/10.1016/10.1016/j.annonc.2023.11.010) have been proposed as therapeutic targets, and novel targeted therapies have been explored in patients with r/r PCNSL in single‐arm phase II trials (see Supplementary Table S9, available at https://doi.org/10.1016/10.1016/j.annonc.2023.11.010). Nevertheless, functional drug screening and next‐generation sequencing assessment for treatment selection in the r/r setting is not recommended outside of prospective trials. The BTK inhibitor ibrutinib, as a single drug at doses of 560‐840 mg/day, has been associated with an ORR of 70%‐77%, a CR rate of 23%‐38% and a median PFS of 4‐5 months in patients with r/r PCNSL or SCNSL.^83^, ^84^ Fungal infection was more frequently observed in patients with r/r PCNSL compared with other types of lymphoma, mostly when ibrutinib was associated with polyChT.^85^ This is probably due in part to prolonged exposure to steroids and impairment of BTK‐dependent fungal immunity and neutrophil function.^85^ Tirabrutinib, a highly selective BTK inhibitor, is a valid option in this setting.^86^

The immunomodulators lenalidomide and pomalidomide, used alone or combined with rituximab, have been evaluated in patients with r/r PCNSL or PVRL with encouraging results (see Supplementary Table S9, available at https://doi.org/10.1016/10.1016/j.annonc.2023.11.010). Although response rates are not particularly high,^75^ lenalidomide has shown good efficacy when used as maintenance and combined with BTK inhibitors.^87^ A phase Ib trial is evaluating rituximab–lenalidomide–ibrutinib in r/r PCNSL (NCT03703167).

Among drugs targeting the phosphoinositide 3‐kinase (PI3K)–mTOR–Akt pathway, temsirolimus has demonstrated good activity but with relevant toxicities, resulting in a 14% TRM rate.^88^ The low activity of buparlisib corresponds with its insufficient concentration in the CSF.^89^ A phase II study evaluating bimiralisib, a dual pan‐PI3K and mTOR inhibitor able to cross the blood–brain barrier, is ongoing (NCT02669511).

The anti‐PD‐1 antibodies nivolumab^90^ and pembrolizumab^91^ are being evaluated in r/r PCNSL. Pembrolizumab 200 mg i.v. was associated with an ORR of 26% and acceptable toxicity.^92^ Results of other trials assessing these immune checkpoint inhibitors in r/r PCNSL are pending [CheckMate 647 (NCT02857426); NCT02779101].

Chimeric antigen receptor T‐cell (CAR‐T) therapy targeting CD19 has demonstrated encouraging results in patients with r/r DLBCL; however, patients with CNS disease were typically excluded from CAR‐T trials due to concerns about severe neurotoxicity. Recently, a phase I/II trial of tisagenlecleucel in 12 patients with r/r PCNSL reported that, at a median follow‐up of 12 months, 6 patients had achieved CR, which was maintained to data cut‐off in 3 patients.^93^ Cytokine release syndrome and immune cell‐associated neurotoxicity syndrome were common but usually of grade 1‐2. A further few cases of PCNSL and SCNSL have been safely and successfully treated with CAR‐T therapy,^94^, ^95^, ^96^ thereby warranting its further investigation.

The use of agents that can permeabilise tumour vessels and increase ChT penetration was recently investigated in patients with r/r PCNSL.^97^, ^98^ Tumour necrosis factor‐α coupled with cysteine–asparagine–glycine–arginine–cysteine–glycine peptide (NGR‐hTNF) targets CD13+ pericytes and endothelial cells of tumour vessels, induces endothelial permeabilisation and improves the tumour access of R–CHOP in r/r PCNSL,^97^ resulting in a reported ORR of 75% and a good toxicity profile.^98^ This treatment was delivered entirely in the outpatient setting, warranting further investigation of NGR‐hTNF in combination with other therapeutic agents in patients with PCNSL.

### Recommendations

- Patients with r/r PCNSL should be registered in a prospective clinical trial assessing novel drugs or strategies [III, A].
- Fit patients with refractory or early relapsed PCNSL can be treated with, for example, HD‐ifosfamide‐ or HD‐AraC‐based combinations, followed by ASCT or WBRT according to previous treatment [III, B].
- Patients with refractory or early relapsed PCNSL unfit for salvage polyChT could be treated with WBRT [V, C] or with a single drug such as ibrutinib, lenalidomide or temozolomide [III, B; not EMA approved, not FDA approved].
- Patients with late relapse of PCNSL could be re‐treated with HD‐MTX, employing the same or similar ChT regimen used in first‐line treatment, and consolidated with ASCT or WBRT in the case of response [IV, B].
- The increased risk of neurotoxicity associated with WBRT should be considered in patients aged >60 years with r/r PCNSL [IV, C].

## FOLLOW‐UP, LONG‐TERM IMPLICATIONS AND SURVIVORSHIP

Given the extended survival of many patients with PCNSL following the widespread use of modern approaches, there is a growing interest in follow‐up and long‐term treatment implications. Notably, combined CRT is associated with disabling neurotoxicity with a cumulative 5‐year incidence rate of 25%‐35%. Prospective data on the optimal follow‐up strategy for patients with PCNSL are lacking. The IPCG guidelines recommend a follow‐up every 3 months for 2 years in patients enrolled in clinical trials, then every 6 months for an additional 3 years and thereafter annually for at least 5 years, for a total of 10 years of follow‐up.^13^ Details on follow‐up strategy and long‐term treatment sequelae are reported in Supplementary Table S10, available at https://doi.org/10.1016/10.1016/j.annonc.2023.11.010.

### Recommendations

- Follow‐up imaging with cranial MRI should be carried out, particularly in patients eligible for intensive salvage therapies, at 3‐month intervals in the first 2 years from the end of treatment, every 6 months for another 3 years and subsequently on an annual basis [IV, B].
- Ophthalmological examination should be carried out annually if not involved initially, and more often if involved initially or in the case of visual deterioration [IV, B].
- CSF assessment should only be carried out in the case of suspected or confirmed relapse [IV, B].
- Neurocognitive function and QoL should be assessed on an annual basis at least within clinical trials [I, B].

## METHODOLOGY

This CPG was developed in accordance with the ESMO standard operating procedures for CPG development (http://www.esmo.org/Guidelines/ESMO-Guidelines-Methodology). The relevant literature has been selected by the expert authors. The FDA/EMA or other regulatory body approval status of new therapies/indications is reported at the time of writing this CPG. Levels of evidence and grades of recommendation have been applied using the system shown in Supplementary Table S11, available at https://doi.org/10.1016/10.1016/j.annonc.2023.11.010. Statements without grading were considered justified standard clinical practice by the authors. For future updates to this CPG, including eUpdates and Living Guidelines, please see the ESMO Guidelines website: https://www.esmo.org/guidelines/guidelines-by-topic/esmo-clinical-practice-guidelines-haematological-malignancies/primary-central-nervous-system-lymphomas.

## AUTHOR CONTRIBUTIONS

All authors conceptualized, wrote, and approved the final version.

## CONFLICT OF INTEREST STATEMENT

**AJMF** reports personal financial interests for advisory board membership for AbbVie, AstraZeneca, Bristol Myers Squibb (BMS), Genmab, Gilead, Incyte, Juno, Novartis, PletixaPharm and Roche; institutional financial interests as local Principal Investigator (PI) for ADC Therapeutics, Amgen, BeiGene, BMS, Genmab, Gilead, Hutchison Medipharma, Incyte, Janssen, Novartis, Pfizer, Pharmacyclics and Takeda; institution research grants from BTG Therapeutics; institutional funding from Roche; non‐financial interests as a member of the Global Outreach Committee of the EHA and as a member of the Board of Directors (President) of Fondazione Italiana Linfomi. **GI** reports personal financial interests as an advisory board member for Gilead, Incyte, Roche and as an invited speaker for Riemser; non‐financial interests as a member of Deutsche Gesellschaft für Hämatologie und Onkologie (DGHO) and a leadership role for the German Lymphoma Alliance (Mitglied des Vorstandes). **JKD** reports personal financial interests as an advisory board member for Eli Lilly. **DPA** reports non‐financial interests as a member of an academic subcommittee for the British Society of Neuroradiologists. **JECB** reports personal financial interests as an advisory board member for Gilead and lecture honorarium from Novartis; institutional financial interests for funding of educational symposia from Roche and TEVA. **TC** reports personal financial interests for advisory board membership and consultancy for Janssen‐Cilag S.p.A; speaking honoraria from Takeda; participation in the Hema for the Future project for Sandoz. **KC** reports personal financial interests as an advisory board member for AbbVie, Atara, Celgene, Incyte, Janssen, Kite, Roche and Takeda; personal financial interests as an invited speaker for Incyte, Kite, Roche and Takeda; non‐financial interests as a member of the American Society of Clinical Oncology (ASCO) and the EHA, and leadership roles with the National Cancer Research Institute (NCRI; chair of UK NCRI T‐cell lymphoma study group). **CPF** reports personal financial interests as an advisory board member for AbbVie, AstraZeneca, Atarabio, BMS, Genmab, Gilead/Kite, Incyte, Janssen, Lilly, MorphoSys, Ono, Roche, SERB and Sobi; personal financial interests as an invited speaker for AbbVie, Gilead/Kite, Incyte, Janssen, Roche and Takeda; institutional financial interests as coordinating PI for BeiGene and Roche; institutional financial interests as a steering committee member for Genmab and MorphoSys; non‐financial interests for an advisory role for Blood Cancer UK (clinical trials funding committee member) and Lymphoma Action (medical advisory panel member); non‐financial interests for leadership roles with Cure Leukaemia (clinical trials steering committee member) and the NCRI (chair of UK NCRI aggressive lymphoma study group). **KHX** reports personal financial interests as an invited speaker for BTG. **MP** reports personal financial interests as an invited speaker for BeiGene and Novartis; personal financial interests for expert testimony for Ventana Roche. **ES** reports personal financial interests as an invited speaker for Riemser Pharma; personal financial interests for a writing engagement for Riemser Pharma; personal financial interests as an advisory board member for SERB Pharmaceuticals; institutional financial interests as a coordinating PI for Riemser Pharma and as a local PI for AbbVie, Riemser Pharma and Roche; non‐financial interests as a PI for AbbVie and Roche; non‐financial interests as a member of the DGHO and German Lymphoma Alliance. **CS** reports institutional funding from AstraZeneca. **LS** reports personal financial interests as an advisory board member for Kyowa Kirin and Takeda; personal financial interests as author royalties from Munksgaard Publishing and Springer Verlag; institutional financial interests as a steering committee member for Varian and ViewRay; non‐financial interests for leadership roles with the International Lymphoma Radiation Oncology Group (Vice Chair) and the Danish Lymphoma Radiation Oncology Group (Chair); non‐financial interests as a PI for the European Organisation for Research and Treatment of Cancer (EORTC); non‐financial interests as a member of ASCO, American Society for Therapeutic Radiology and Oncology (ASTRO) and European Society for Therapeutic Radiology and Oncology (ESTRO). **EZ** reports personal financial interests as an advisory board member for AbbVie, BeiGene, BMS, Curis, Eli Lilly, Incyte, Ipsen, Janssen, Merck, Miltenyi Biomedicine and Roche; institutional financial interests for travel grants from BeiGene, Gilead, Janssen and Roche; institutional financial interests for trial sponsorship from AstraZeneca, BeiGene, Celgene/BMS, Incyte, Janssen and Roche. **CB** reports personal financial interests as an invited speaker for AbbVie, Pfizer and Sobi; personal financial interests as an advisory board member for BeiGene, Celltrion, Gilead Sciences, Incyte, Janssen, MorphoSys, Novartis, Regeneron and Roche; institutional funding from AbbVie, Amgen, Celltrion, Janssen, MSD, Pfizer and Roche. **MJ** reports personal financial interests as an advisory board member for BMS, Genmab, Gilead, Janssen and Novartis; personal financial interests as an invited speaker for Incyte; institutional financial interests as an invited speaker for Roche; institutional funding from AbbVie, AstraZeneca, Celgene, Gilead, Janssen and Roche; institutional financial interests as coordinating PI for BioInvent. **MD** reports personal financial interests as an invited speaker for AstraZeneca, Gilead/Kite, Janssen, Novartis and Roche; personal financial interests as an advisory board member for AstraZeneca, BeiGene, BMS/Celgene, Genmab, Gilead, Janssen, Lilly/Loxo, Novartis and Roche; institutional research grants from AbbVie, Bayer, Celgene, Janssen and Roche; institutional funding from Gilead/Kite; non‐financial interests as a member of ASCO, American Society of Hematology (ASH; subcommittee member), DGHO (prior Board member), EHA (Executive Board member), ESMO (Faculty member) and Lymphoma Research Foundation [LRF; Mantle Cell Lymphoma (MCL) Consortium member]. **DM** has declared no conflicts of interest.

## FUNDING

No external funding has been received for the preparation of this guideline. Production costs have been covered by ESMO (for Annals of Oncology) and EHA (for HemaSphere) central funds.

## Supporting information

Supporting information.

## Data Availability

Data sharing is not applicable to this article as no new data were created or analyzed in this study.

## References

[1] Kluin PM , Deckert M , Ferry JA. Primary diffuse large B‐cell lymphoma of the CNS. In: Swerdlow SH , Campo E , Harris NL , et al. eds. WHO Classification of Tumours of Haematopoietic and Lymphoid Tissues. 4th ed. Lyon, France: IARC; 2017:300‐302.

[2] Alaggio R , Amador C , Anagnostopoulos I , et al. The 5th edition of the World Health Organization Classification of Haematolymphoid Tumours: lymphoid neoplasms. Leukemia. 2022;36(7):1720‐1748. 35732829 10.1038/s41375-022-01620-2 PMC9214472

[3] Campo E , Jaffe ES , Cook JR , et al. The International Consensus Classification of Mature Lymphoid Neoplasms: a report from the Clinical Advisory Committee. Blood. 2022;140(11):1229‐1253. 35653592 10.1182/blood.2022015851 PMC9479027

[4] Deckert M , Ferry JA. Primary diffuse large B‐cell lymphoma of the CNS. In: WHO Classification of Tumours Editorial Board, ed. WHO Classification of Tumours: Central Nervous System Tumours. 5th ed Lyon, France: IARC; 2021:351‐355.

[5] Engels EA , Biggar RJ , Hall HI , et al. Cancer risk in people infected with human immunodeficiency virus in the United States. Int J Cancer. 2008;123(1):187‐194. 18435450 10.1002/ijc.23487

[6] Villano JL , Koshy M , Shaikh H , et al. Age, gender, and racial differences in incidence and survival in primary CNS lymphoma. Br J Cancer. 2011;105(9):1414‐1418. 21915121 10.1038/bjc.2011.357 PMC3241537

[7] Houillier C , Soussain C , Ghesquieres H , et al. Management and outcome of primary CNS lymphoma in the modern era: an LOC network study. Neurology. 2020;94(10):e1027‐e1039. 31907289 10.1212/WNL.0000000000008900 PMC7238921

[8] Ferreri AJ , Reni M , Pasini F , et al. A multicenter study of treatment of primary CNS lymphoma. Neurology. 2002;58(10):1513‐1520. 12034789 10.1212/wnl.58.10.1513

[9] Soussain C , Malaise D , Cassoux N. Primary vitreoretinal lymphoma: a diagnostic and management challenge. Blood. 2021;138(17):1519‐1534. 34036310 10.1182/blood.2020008235

[10] Miserocchi E , Ferreri AJM , Giuffre C , et al. MYD88 L265P mutation detection in the aqueous humor of patients with vitreoretinal lymphoma. Retina. 2019;39(4):679‐684. 30204732 10.1097/IAE.0000000000002319

[11] Bonzheim I , Giese S , Deuter C , et al. High frequency of MYD88 mutations in vitreoretinal B‐cell lymphoma: a valuable tool to improve diagnostic yield of vitreous aspirates. Blood. 2015;126(1):76‐79. 25900979 10.1182/blood-2015-01-620518

[12] Barajas RF , Politi LS , Anzalone N , et al. Consensus recommendations for MRI and PET imaging of primary central nervous system lymphoma: guideline statement from the International Primary CNS Lymphoma Collaborative Group (IPCG). Neuro Oncol. 2021;23(7):1056‐1071. 33560416 10.1093/neuonc/noab020 PMC8248856

[13] Abrey LE , Batchelor TT , Ferreri AJ , et al. Report of an international workshop to standardize baseline evaluation and response criteria for primary CNS lymphoma. J Clin Oncol. 2005;23(22):5034‐5043. 15955902 10.1200/JCO.2005.13.524

[14] Ferreri AJM , Calimeri T , Lopedote P , et al. MYD88 L265P mutation and interleukin‐10 detection in cerebrospinal fluid are highly specific discriminating markers in patients with primary central nervous system lymphoma: results from a prospective study. Br J Haematol. 2021;193(3):497‐505. 33620087 10.1111/bjh.17357

[15] Hegde U , Filie A , Little RF , et al. High incidence of occult leptomeningeal disease detected by flow cytometry in newly diagnosed aggressive B‐cell lymphomas at risk for central nervous system involvement: the role of flow cytometry versus cytology. Blood. 2005;105(2):496‐502. 15358629 10.1182/blood-2004-05-1982

[16] Ferreri AJM , Doorduijn JK , Re A , et al. MATRix‐RICE therapy and autologous haematopoietic stem‐cell transplantation in diffuse large B‐cell lymphoma with secondary CNS involvement (MARIETTA): an international, single‐arm, phase 2 trial. Lancet Haematol. 2021;8(2):e110‐e121. 33513372 10.1016/S2352-3026(20)30366-5 PMC7844712

[17] Ferreri AJ , Blay JY , Reni M , et al. Prognostic scoring system for primary CNS lymphomas: the International Extranodal Lymphoma Study Group experience. J Clin Oncol. 2003;21(2):266‐272. 12525518 10.1200/JCO.2003.09.139

[18] Abrey LE , Ben‐Porat L , Panageas KS , et al. Primary central nervous system lymphoma: the Memorial Sloan‐Kettering Cancer Center prognostic model. J Clin Oncol. 2006;24(36):5711‐5715. 17116938 10.1200/JCO.2006.08.2941

[19] Correa DD , Maron L , Harder H , et al. Cognitive functions in primary central nervous system lymphoma: literature review and assessment guidelines. Ann Oncol. 2007;18(7):1145‐1151. 17284616 10.1093/annonc/mdl464

[20] Mendez JS , Ostrom QT , Gittleman H , et al. The elderly left behind‐changes in survival trends of primary central nervous system lymphoma over the past 4 decades. Neuro Oncol. 2018;20(5):687‐694. 29036697 10.1093/neuonc/nox187 PMC5892148

[21] Mead GM , Bleehen NM , Gregor A , et al. A medical research council randomized trial in patients with primary cerebral non‐Hodgkin lymphoma: cerebral radiotherapy with and without cyclophosphamide, doxorubicin, vincristine, and prednisone chemotherapy. Cancer. 2000;89(6):1359‐1370. 11002232

[22] Batchelor T , Carson K , O'Neill A , et al. Treatment of primary CNS lymphoma with methotrexate and deferred radiotherapy: a report of NABTT 96‐07. J Clin Oncol. 2003;21(6):1044‐1049. 12637469 10.1200/JCO.2003.03.036

[23] Ghesquieres H , Ferlay C , Sebban C , et al. Long‐term follow‐up of an age‐adapted C5R protocol followed by radiotherapy in 99 newly diagnosed primary CNS lymphomas: a prospective multicentric phase II study of the Groupe d'Etude des Lymphomes de l'Adulte (GELA). Ann Oncol. 2010;21(4):842‐850. 19914958 10.1093/annonc/mdp529

[24] Ferreri AJ , Reni M , Foppoli M , et al. High‐dose cytarabine plus high‐dose methotrexate versus high‐dose methotrexate alone in patients with primary CNS lymphoma: a randomised phase 2 trial. Lancet. 2009;374(9700):1512‐1520. 19767089 10.1016/S0140-6736(09)61416-1

[25] Ferreri AJ , Cwynarski K , Pulczynski E , et al. Chemoimmunotherapy with methotrexate, cytarabine, thiotepa, and rituximab (MATRix regimen) in patients with primary CNS lymphoma: results of the first randomisation of the International Extranodal Lymphoma Study Group‐32 (IELSG32) phase 2 trial. Lancet Haematol. 2016;3(5):e217‐e227. 27132696 10.1016/S2352-3026(16)00036-3

[26] Ferreri AJM , Cwynarski K , Pulczynski E , et al. Long‐term efficacy, safety and neurotolerability of MATRix regimen followed by autologous transplant in primary CNS lymphoma: 7‐year results of the IELSG32 randomized trial. Leukemia. 2022;36(7):1870‐1878. 35562406 10.1038/s41375-022-01582-5

[27] Schorb E , Fox CP , Kasenda B , et al. Induction therapy with the MATRix regimen in patients with newly diagnosed primary diffuse large B‐cell lymphoma of the central nervous system ‐ an international study of feasibility and efficacy in routine clinical practice. Br J Haematol. 2020;189(5):879‐887. 31997308 10.1111/bjh.16451

[28] Bromberg JEC , Issa S , Bakunina K , et al. Rituximab in patients with primary CNS lymphoma (HOVON 105/ALLG NHL 24): a randomised, open‐label, phase 3 intergroup study. Lancet Oncol. 2019;20(2):216‐228. 30630772 10.1016/S1470-2045(18)30747-2

[29] Houillier C , Taillandier L , Dureau S , et al. Radiotherapy or autologous stem‐cell transplantation for primary CNS lymphoma in patients 60 years of age and younger: results of the Intergroup ANOCEF‐GOELAMS randomized phase II PRECIS study. J Clin Oncol. 2019;37(10):823‐833. 30785830 10.1200/JCO.18.00306

[30] Houillier C , Dureau S , Taillandier L , et al. Radiotherapy or autologous stem‐cell transplantation for primary CNS lymphoma in patients age 60 years and younger: long‐term results of the randomized phase II PRECIS study. J Clin Oncol. 2022;40(32):3692‐3698. 35834762 10.1200/JCO.22.00491

[31] Omuro AMP , DeAngelis LM , Karrison T , et al. Randomized phase II study of rituximab, methotrexate (MTX), procarbazine, vincristine, and cytarabine (R‐MPV‐A) with and without low‐dose whole‐brain radiotherapy (LD‐WBRT) for newly diagnosed primary CNS lymphoma (PCNSL). J Clin Oncol. 2020;38(15_suppl):2501.

[32] Morris PG , Correa DD , Yahalom J , et al. Rituximab, methotrexate, procarbazine, and vincristine followed by consolidation reduced‐dose whole‐brain radiotherapy and cytarabine in newly diagnosed primary CNS lymphoma: final results and long‐term outcome. J Clin Oncol. 2013;31(31):3971‐3979. 24101038 10.1200/JCO.2013.50.4910 PMC5569679

[33] Schmitt AM , Herbrand AK , Fox CP , et al. Rituximab in primary central nervous system lymphoma‐a systematic review and meta‐analysis. Hematol Oncol. 2019;37(5):548‐557. 31418878 10.1002/hon.2666

[34] Khan RB , Shi W , Thaler HT , et al. Is intrathecal methotrexate necessary in the treatment of primary CNS lymphoma? J Neurooncol. 2002;58(2):175‐178. 12164690 10.1023/a:1016077907952

[35] Ferreri AJM , Cwynarski K , Pulczynski E , et al. Whole‐brain radiotherapy or autologous stem‐cell transplantation as consolidation strategies after high‐dose methotrexate‐based chemoimmunotherapy in patients with primary CNS lymphoma: results of the second randomisation of the International Extranodal Lymphoma Study Group‐32 phase 2 trial. Lancet Haematol. 2017;4(11):e510‐e523. 29054815 10.1016/S2352-3026(17)30174-6

[36] Abrey LE , Moskowitz CH , Mason WP , et al. Intensive methotrexate and cytarabine followed by high‐dose chemotherapy with autologous stem‐cell rescue in patients with newly diagnosed primary CNS lymphoma: an intent‐to‐treat analysis. J Clin Oncol. 2003;21(22):4151‐4156. 14615443 10.1200/JCO.2003.05.024

[37] Scordo M , Wang TP , Ahn KW , et al. Outcomes associated with thiotepa‐based conditioning in patients with primary central nervous system lymphoma after autologous hematopoietic cell transplant. JAMA Oncol. 2021;7(7):993‐1003. 33956047 10.1001/jamaoncol.2021.1074 PMC8283558

[38] Illerhaus G , Marks R , Ihorst G , et al. High‐dose chemotherapy with autologous stem‐cell transplantation and hyperfractionated radiotherapy as first‐line treatment of primary CNS lymphoma. J Clin Oncol. 2006;24(24):3865‐3870. 16864853 10.1200/JCO.2006.06.2117

[39] Illerhaus G , Kasenda B , Ihorst G , et al. High‐dose chemotherapy with autologous haemopoietic stem cell transplantation for newly diagnosed primary CNS lymphoma: a prospective, single‐arm, phase 2 trial. Lancet Haematol. 2016;3(8):e388‐e397. 27476790 10.1016/S2352-3026(16)30050-3

[40] Omuro A , Correa DD , DeAngelis LM , et al. R‐MPV followed by high‐dose chemotherapy with TBC and autologous stem‐cell transplant for newly diagnosed primary CNS lymphoma. Blood. 2015;125(9):1403‐1410. 25568347 10.1182/blood-2014-10-604561 PMC4342354

[41] Schorb E , Fox CP , Fritsch K , et al. High‐dose thiotepa‐based chemotherapy with autologous stem cell support in elderly patients with primary central nervous system lymphoma: a European retrospective study. Bone Marrow Transplant. 2017;52(8):1113‐1119. 28436974 10.1038/bmt.2017.23

[42] Schorb E , Kasenda B , Ihorst G , et al. High‐dose chemotherapy and autologous stem cell transplant in elderly patients with primary CNS lymphoma: a pilot study. Blood Adv. 2020;4(14):3378‐3381. 32722778 10.1182/bloodadvances.2020002064 PMC7391148

[43] Fritsch K , Kasenda B , Schorb E , et al. High‐dose methotrexate‐based immuno‐chemotherapy for elderly primary CNS lymphoma patients (PRIMAIN study). Leukemia. 2017;31(4):846‐852. 27843136 10.1038/leu.2016.334 PMC5383936

[44] Schorb E , Isbell L , Kerkhoff A , et al. High‐dose chemotherapy and autologous stem cell transplant in elderly and fit primary CNS lymphoma patients – a multicenter study by the Cooperative PCNSL study group (MARTA study). Blood. 2022;140(suppl 1):1773‐1774.

[45] Correa DD , DeAngelis LM , Shi W , et al. Cognitive functions in survivors of primary central nervous system lymphoma. Neurology. 2004;62(4):548‐555. 14981169 10.1212/01.wnl.0000109673.75316.d8

[46] Fisher B , Seiferheld W , Schultz C , et al. Secondary analysis of Radiation Therapy Oncology Group study (RTOG) 9310: an intergroup phase II combined modality treatment of primary central nervous system lymphoma. J Neurooncol. 2005;74(2):201‐205. 16193393 10.1007/s11060-004-6596-9

[47] Thiel E , Korfel A , Martus P , et al. High‐dose methotrexate with or without whole brain radiotherapy for primary CNS lymphoma (G‐PCNSL‐SG‐1): a phase 3, randomised, non‐inferiority trial. Lancet Oncol. 2010;11(11):1036‐1047. 20970380 10.1016/S1470-2045(10)70229-1

[48] Lai R , Rosenblum MK , DeAngelis LM. Primary CNS lymphoma: a whole‐brain disease? Neurology. 2002;59(10):1557‐1562. 12451197 10.1212/01.wnl.0000034256.20173.ea

[49] Shibamoto Y , Hayabuchi N , Hiratsuka J , et al. Is whole‐brain irradiation necessary for primary central nervous system lymphoma? Patterns of recurrence after partial‐brain irradiation. Cancer. 2003;97(1):128‐133. 12491514 10.1002/cncr.11035

[50] Ferreri AJ , Abrey LE , Blay JY , et al. Summary statement on primary central nervous system lymphomas from the Eighth International Conference on Malignant Lymphoma, Lugano, Switzerland, June 12 to 15, 2002. J Clin Oncol. 2003;21(12):2407‐2414. 12805341 10.1200/JCO.2003.01.135

[51] Yahalom J , Illidge T , Specht L , et al. Modern radiation therapy for extranodal lymphomas: field and dose guidelines from the International Lymphoma Radiation Oncology Group. Int J Radiat Oncol Biol Phys. 2015;92(1):11‐31. 25863750 10.1016/j.ijrobp.2015.01.009

[52] Alvarez‐Pinzon AM , Wolf AL , Swedberg H , et al. Primary central nervous system lymphoma (PCNSL): analysis of treatment by gamma knife radiosurgery and chemotherapy in a prospective, observational study. Cureus. 2016;8(7):e697. 27570717 10.7759/cureus.697 PMC4996544

[53] Rubenstein JL , Hsi ED , Johnson JL , et al. Intensive chemotherapy and immunotherapy in patients with newly diagnosed primary CNS lymphoma: CALGB 50202 (Alliance 50202). J Clin Oncol. 2013;31(25):3061‐3068. 23569323 10.1200/JCO.2012.46.9957 PMC3753699

[54] Batchelor T , Giri S , Ruppert AS , et al. Myeloablative versus non‐myeloablative consolidative chemotherapy for newly diagnosed primary central nervous system lymphoma: results of CALGB 51101 (Alliance). J Clin Oncol. 2021;39(15_suppl):7506.

[55] Illerhaus G , Ferreri AJM , Binder M , et al. Effects on survival of non‐myeloablative chemoimmunotherapy compared to high‐dose chemotherapy followed by autologous stem cell transplantation (HDC‐ASCT) as consolidation therapy in patients with primary CNS lymphoma ‐ results of an international randomized phase III trial (MATRix/IELSG43). Blood. 2022;140(suppl 2). LBA‐3.

[56] Pulczynski EJ , Kuittinen O , Erlanson M , et al. Successful change of treatment strategy in elderly patients with primary central nervous system lymphoma by de‐escalating induction and introducing temozolomide maintenance: results from a phase II study by the Nordic Lymphoma Group. Haematologica. 2015;100(4):534‐540. 25480497 10.3324/haematol.2014.108472 PMC4380727

[57] Mishima K , Nishikawa R , Narita Y , et al. Randomized phase III study of high‐dose methotrexate and whole‐brain radiotherapy with/without temozolomide for newly diagnosed primary CNS lymphoma: JCOG1114C. Neuro Oncol. 2023;25(4):687‐698. 36334050 10.1093/neuonc/noac246 PMC10076938

[58] Welch MR , Omuro A , Deangelis LM. Outcomes of the oldest patients with primary CNS lymphoma treated at Memorial Sloan‐Kettering Cancer Center. Neuro Oncol. 2012;14(10):1304‐1311. 22952196 10.1093/neuonc/nos207 PMC3452344

[59] Martinez‐Calle N , Poynton E , Alchawaf A , et al. Outcomes of older patients with primary central nervous system lymphoma treated in routine clinical practice in the UK: methotrexate dose intensity correlates with response and survival. Br J Haematol. 2020;190(3):394‐404. 32232989 10.1111/bjh.16592

[60] Fox CP , Phillips EH , Smith J , et al. Guidelines for the diagnosis and management of primary central nervous system diffuse large B‐cell lymphoma. Br J Haematol. 2019;184(3):348‐363. 30467845 10.1111/bjh.15661

[61] Kasenda B , Ferreri AJ , Marturano E , et al. First‐line treatment and outcome of elderly patients with primary central nervous system lymphoma (PCNSL)–a systematic review and individual patient data meta‐analysis. Ann Oncol. 2015;26(7):1305‐1313. 25701456 10.1093/annonc/mdv076 PMC4735103

[62] Illerhaus G , Marks R , Muller F , et al. High‐dose methotrexate combined with procarbazine and CCNU for primary CNS lymphoma in the elderly: results of a prospective pilot and phase II study. Ann Oncol. 2009;20(2):319‐325. 18953065 10.1093/annonc/mdn628

[63] Grimm SA , Pulido JS , Jahnke K , et al. Primary intraocular lymphoma: an International Primary Central Nervous System Lymphoma Collaborative Group Report. Ann Oncol. 2007;18(11):1851‐1855. 17804469 10.1093/annonc/mdm340

[64] Hoang‐Xuan K , Taillandier L , Chinot O , et al. Chemotherapy alone as initial treatment for primary CNS lymphoma in patients older than 60 years: a multicenter phase II study (26952) of the European Organization for Research and Treatment of Cancer Brain Tumor Group. J Clin Oncol. 2003;21(14):2726‐2731. 12860951 10.1200/JCO.2003.11.036

[65] Houillier C , Ghesquieres H , Chabrot C , et al. Rituximab, methotrexate, procarbazine, vincristine and intensified cytarabine consolidation for primary central nervous system lymphoma (PCNSL) in the elderly: a LOC network study. J Neurooncol. 2017;133(2):315‐320. 28432587 10.1007/s11060-017-2435-7

[66] Grimm SA , McCannel CA , Omuro AMP , et al. Primary CNS lymphoma with intraocular involvement: International PCNSL Collaborative Group Report. Neurology. 2008;71(17):1355‐1360. 18936428 10.1212/01.wnl.0000327672.04729.8c PMC4109164

[67] Birsen R , Willems L , Pallud J , et al. Efficacy and safety of high‐dose etoposide cytarabine as consolidation following rituximab methotrexate temozolomide induction in newly diagnosed primary central nervous system lymphoma in immunocompetent patients. Haematologica. 2018;103(7):e296‐e299. 29472354 10.3324/haematol.2017.185843 PMC6029551

[68] Hoang‐Xuan K , Chinot OL , Taillandier L. Treatment of primary central nervous system lymphoma in the elderly. Semin Oncol. 2003;30(6 suppl 19):53‐57. 14765387 10.1053/j.seminoncol.2003.11.032

[69] Vu K , Mannis G , Hwang J , et al. Low‐dose lenalidomide maintenance after induction therapy in older patients with primary central nervous system lymphoma. Br J Haematol. 2019;186(1):180‐183. 30714128 10.1111/bjh.15787 PMC8776513

[70] Laack NN , Ballman KV , Brown PB , et al. Whole‐brain radiotherapy and high‐dose methylprednisolone for elderly patients with primary central nervous system lymphoma: results of North Central Cancer Treatment Group (NCCTG) 96‐73‐51. Int J Radiat Oncol Biol Phys. 2006;65(5):1429‐1439. 16863926 10.1016/j.ijrobp.2006.03.061

[71] Hormigo A , Abrey L , Heinemann MH , et al. Ocular presentation of primary central nervous system lymphoma: diagnosis and treatment. Br J Haematol. 2004;126(2):202‐208. 15238140 10.1111/j.1365-2141.2004.05028.x

[72] de la Fuente MI , Alderuccio JP , Reis IM , et al. Bilateral radiation therapy followed by methotrexate‐based chemotherapy for primary vitreoretinal lymphoma. Am J Hematol. 2019;94(4):455‐460. 30663807 10.1002/ajh.25414

[73] Lam M , Touitou V , Choquet S , et al. Intravenous high‐dose methotrexate based systemic therapy in the treatment of isolated primary vitreoretinal lymphoma: an LOC network study. Am J Hematol. 2021;96(7):823‐833. 33864703 10.1002/ajh.26199

[74] Soussain C , Suzan F , Hoang‐Xuan K , et al. Results of intensive chemotherapy followed by hematopoietic stem‐cell rescue in 22 patients with refractory or recurrent primary CNS lymphoma or intraocular lymphoma. J Clin Oncol. 2001;19(3):742‐749. 11157026 10.1200/JCO.2001.19.3.742

[75] Ghesquieres H , Chevrier M , Laadhari M , et al. Lenalidomide in combination with intravenous rituximab (REVRI) in relapsed/refractory primary CNS lymphoma or primary intraocular lymphoma: a multicenter prospective ‘proof of concept’ phase II study of the French Oculo‐Cerebral lymphoma (LOC) Network and the Lymphoma Study Association (LYSA). Ann Oncol. 2019;30(4):621‐628. 30698644 10.1093/annonc/mdz032

[76] Ambady P , Holdhoff M , Bonekamp D , et al. Late relapses in primary CNS lymphoma after complete remissions with high‐dose methotrexate monotherapy. CNS Oncol. 2015;4(6):393‐398. 26507609 10.2217/cns.15.34 PMC4834699

[77] Ambady P , Fu R , Netto JP , et al. Patterns of relapse in primary central nervous system lymphoma: inferences regarding the role of the neuro‐vascular unit and monoclonal antibodies in treating occult CNS disease. Fluids Barriers CNS. 2017;14(1):16. 28577579 10.1186/s12987-017-0064-3 PMC5457655

[78] Jahnke K , Thiel E , Martus P , et al. Relapse of primary central nervous system lymphoma: clinical features, outcome and prognostic factors. J Neurooncol. 2006;80(2):159‐165. 16699873 10.1007/s11060-006-9165-6

[79] Langner‐Lemercier S , Houillier C , Soussain C , et al. Primary CNS lymphoma at first relapse/progression: characteristics, management, and outcome of 256 patients from the French LOC network. Neuro Oncol. 2016;18(9):1297‐1303. 26951382 10.1093/neuonc/now033 PMC4998995

[80] Mappa S , Marturano E , Licata G , et al. Salvage chemoimmunotherapy with rituximab, ifosfamide and etoposide (R‐IE regimen) in patients with primary CNS lymphoma relapsed or refractory to high‐dose methotrexate‐based chemotherapy. Hematol Oncol. 2013;31(3):143‐150. 23161567 10.1002/hon.2037

[81] Plotkin SR , Betensky RA , Hochberg FH , et al. Treatment of relapsed central nervous system lymphoma with high‐dose methotrexate. Clin Cancer Res. 2004;10(17):5643‐5646. 15355887 10.1158/1078-0432.CCR-04-0159

[82] Nguyen PL , Chakravarti A , Finkelstein DM , et al. Results of whole‐brain radiation as salvage of methotrexate failure for immunocompetent patients with primary CNS lymphoma. J Clin Oncol. 2005;23(7):1507‐1513. 15735126 10.1200/JCO.2005.01.161

[83] Soussain C , Choquet S , Blonski M , et al. Ibrutinib monotherapy for relapse or refractory primary CNS lymphoma and primary vitreoretinal lymphoma: final analysis of the phase II ‘proof‐of‐concept’ iLOC study by the Lymphoma study association (LYSA) and the French oculo‐cerebral lymphoma (LOC) network. Eur J Cancer. 2019;117:121‐130. 31279304 10.1016/j.ejca.2019.05.024

[84] Grommes C , Gavrilovic IT , Kaley TJ , et al. Updated results of single‐agent ibrutinib in recurrent/refractory primary (PCNSL) and secondary CNS lymphoma (SCNSL). J Clin Oncol. 2017;35(15_suppl):7515.

[85] Lionakis MS , Dunleavy K , Roschewski M , et al. Inhibition of B cell receptor signaling by ibrutinib in primary CNS lymphoma. Cancer Cell. 2017;31(6):833‐843. 28552327 10.1016/j.ccell.2017.04.012 PMC5571650

[86] Narita Y , Nagane M , Mishima K , et al. Phase I/II study of tirabrutinib, a second‐generation Bruton's tyrosine kinase inhibitor, in relapsed/refractory primary central nervous system lymphoma. Neuro Oncol. 2021;23(1):122‐133. 32583848 10.1093/neuonc/noaa145 PMC7850159

[87] Houillier C , Chabrot CM , Moles‐Moreau MP , et al. Rituximab‐lenalidomide‐ibrutinib combination for relapsed/refractory primary CNS lymphoma: a case series of the LOC network. Neurology. 2021;97(13):628‐631. 34580183 10.1212/WNL.0000000000012515

[88] Korfel A , Schlegel U , Herrlinger U , et al. Phase II trial of temsirolimus for relapsed/refractory primary CNS lymphoma. J Clin Oncol. 2016;34(15):1757‐1763. 26976424 10.1200/JCO.2015.64.9897

[89] Grommes C , Pentsova E , Nolan C , et al. Phase II study of single agent buparlisib in recurrent/refractory primary (PCNSL) and secondary CNS lymphoma (SCNSL). Ann Oncol. 2016;27(suppl 6):vi106.

[90] Nayak L , Iwamoto FM , LaCasce A , et al. PD‐1 blockade with nivolumab in relapsed/refractory primary central nervous system and testicular lymphoma. Blood. 2017;129(23):3071‐3073. 28356247 10.1182/blood-2017-01-764209 PMC5766844

[91] Ambady P , Szidonya L , Firkins J , et al. Combination immunotherapy as a non‐chemotherapy alternative for refractory or recurrent CNS lymphoma. Leuk Lymphoma. 2019;60(2):515‐518. 30033836 10.1080/10428194.2018.1480771 PMC6342672

[92] Hoang‐Xuan K , Houot R , Soussain C , et al. First results of the Acsé pembrolizumab phase II in the primary CNS lymphoma (PCNSL) cohort. Blood. 2020;136(suppl 1):15‐16.

[93] Frigault MJ , Dietrich J , Gallagher K , et al. Safety and efficacy of tisagenlecleucel in primary CNS lymphoma: a phase 1/2 clinical trial. Blood. 2022;139(15):2306‐2315. 35167655 10.1182/blood.2021014738 PMC9012129

[94] Tu S , Zhou X , Guo Z , et al. CD19 and CD70 dual‐target chimeric antigen receptor T‐cell therapy for the treatment of relapsed and refractory primary central nervous system diffuse large B‐cell lymphoma. Front Oncol. 2019;9:1350. 31867275 10.3389/fonc.2019.01350 PMC6904344

[95] Alcantara M , Houillier C , Blonski M , et al. CAR T‐cell therapy in primary central nervous system lymphoma: the clinical experience of the French LOC network. Blood. 2022;139(5):792‐796. 34871363 10.1182/blood.2021012932 PMC8814680

[96] Frigault MJ , Dietrich J , Martinez‐Lage M , et al. Tisagenlecleucel CAR T‐cell therapy in secondary CNS lymphoma. Blood. 2019;134(11):860‐866. 31320380 10.1182/blood.2019001694 PMC7022436

[97] Ferreri AJM , Calimeri T , Conte GM , et al. R‐CHOP preceded by blood‐brain barrier permeabilization with engineered tumor necrosis factor‐alpha in primary CNS lymphoma. Blood. 2019;134(3):252‐262. 31118164 10.1182/blood.2019000633

[98] Ferreri AJM , Calimeri T , Ponzoni M , et al. Improving the antitumor activity of R‐CHOP with NGR‐hTNF in primary CNS lymphoma: final results of a phase 2 trial. Blood Adv. 2020;4(15):3648‐3658. 32766857 10.1182/bloodadvances.2020002270 PMC7422104

