# Correction to “Primary central nervous system lymphomas: EHA–ESMO Clinical Practice Guideline for diagnosis, treatment and follow‐up”

**DOI:** 10.1002/hem3.118

**Published:** 2024-07-22

Ferreri AJM, Illerhaus G, Doorduijn JK, et al. Primary central nervous system lymphomas: EHA–ESMO Clinical Practice Guideline for diagnosis, treatment and follow‐up. *HemaSphere*. 2024;8:e89.

In the author listing of this manuscript, the first name of the first author was misspelled. The correct spelling is Andrés JM Ferreri.

The original article has been corrected. We apologize for this error.

