# Supplementary material for: Primary central nervous system lymphomas: EHA–ESMO Clinical Practice Guideline for diagnosis, treatment and follow‐up
Source: Hemasphere. 2024 Jun 4;8(6):e89. doi: 10.1002/hem3.89 (PMC11148853; doi:10.1002/hem3.89)
Supplement: Supplementary file 1 — Supporting information. [file HEM3-8-e89-s001.pdf]

**Primary central nervous system lymphomas: EHA–ESMO Clinical Practice Guideline for diagnosis, treatment and follow-up**

**SUPPLEMENTARY MATERIAL**

**Supplementary Table S1. 3T MRI protocol for PCNSL assessment recommended by the IPCG[1]**

| | **DWI** | **T1W-Pre[a]** | **T2W** | **DSC perfusion[b,c,d]** **Contrast injection[b]** | **CE-T2W-FLAIR** | **T1W-Post[a,e]** |
|---|---|---|---|---|---|---|
| Sequence | SS-EPI[f] | TSE[g] | TSE[g] | GE-EPI | TSE[g] | TSE[g] |
| Plane | Axial | Any | Any | Axial | Any | Any |
| Mode | 2D | 3D | 3D | 2D | 3D | 3D[h] |
| TR (ms) | >5000 | 550-750 | >2500 | 1000-1500 | >6000 | 550-750 |
| TE (ms) | Min | Min | 80-120 | 20-35 | 90-140 | Min |
| TI (ms) | | | | | 2000-2500 | |
| Flip angle (degree) | 90/180 | Default[i] | 90/≥160 | 30-35 | 90/≥160 | Default[i] |
| Frequency | 128 | 256 | ≥256 | ≥96 | ≥256 | 256 |
| Phase | 128 | 256 | ≥256 | ≥96 | ≥256 | 256 |
| NEX | ≥1 | ≥1 | ≥1 | 1 | ≥1 | ≥1 |
| FOV (mm) | 240 | 256 | 240 | 240 | 240 | 256 |
| Slice thickness (mm) | 3 | 1 | 1 | 3-5 as needed to cover tumour | 1 | 1 |

| | | | | | | |
|---|---|---|---|---|---|---|
| Spacing (mm) | 0 | 0 | 0 | 0-1 as needed to cover tumour | 0 | 0 |
| Other options | *b* = 0, 500 and 1000 s/mm$^2$, ≥3 directions | Consider fat saturation | | 30-60 pre-bolus time points; >120 total time points; centred on tumour. DCE is optional before DSC | Consider fat saturation | Consider fat saturation |
| Parallel imaging[j] | Up to 2x | Up to 2x | Up to 2x | Up to 2x | Up to 2x | Up to 2x |
| Estimated time (min)[k] | 2-4 | 5-8 | 5-8 | 2-4 | 5-8 | 5-8 |

ADC, apparent diffusion coefficient; CAIPI, controlled aliasing in parallel imaging; CE-T2W-FLAIR, contrast-enhanced T2-weighted fluid-attenuated inversion recovery; DCE, dynamic contrast-enhanced; DSC, dynamic susceptibility contrast; DWI, diffusion-weighted imaging; EPI, echo-planar imaging; FLAIR, fluid-attenuated inversion-recovery; FOV, field of view; FSE, fast spin echo; GE-EPI, gradient echo echo-planar imaging; GRAPPA, generalised autocalibrating partially parallel acquisition; iPAT, integrated parallel acquisition technique; IPCG, International PCNSL Collaborative Group; MR, magnetic resonance; MRI, magnetic resonance imaging; NEX, number of excitations; PCNSL, primary central nervous system lymphoma; SMS, simultaneous multi-slice; SS-EPI, single-shot echo-planar imaging; T1-W, T1-weighted; T2-W, T2-weighted; TE, time to echo; TI, inversion time; TR, repetition time; TSE, turbo spin echo.

[a]Postcontrast 3D T1-W images should be collected with equivalent parameters to precontrast 3D TI-W images.

[b]0.1 mmol/kg dose injection with a gadolinium-chelated contrast agent as a single total dose is recommended. For DSC perfusion, contrast injection is carried out after obtaining 30-50 DSC time points. In the absence of carrying out DCE, no DSC preload contrast dose is recommended given use of low flip angle. DSC perfusion can be carried out with the 'ideal' protocol at 3T as well as with the 'minimum standard' protocols at 3T and 1.5T. The use of a power injector is desirable at an injection rate of 3-5 cc/sec.

[c]If both DCE and DSC acquisitions are desired and carried out on 3T unit, the 0.1 mmol/kg (single total dose) can be split into two separate half doses (½ + ½) over two sequential injections. Alternatively, for clinical sites that employ a double-dosing protocol, a 0.2 mmol/kg (double total dose) can be split into two separate single doses (1 + 1) over two sequential injections. For both dosing protocols, DCE will be acquired during the first injection, and DSC will be acquired during the second injection. However, for the (1 + 1) dose schema, the post-contrast T1-W image should be acquired after DCE and before DSC, per the standardised DSC recommendations for high-grade gliomas.

[d]If only DCE acquisition is desired, the DCE sequence will replace the DSC and employ the full single dose (0.1 mmol/kg) contrast injection.

[e]3D post-contrast T1-W images are collected between 4 and 8 minutes after contrast injection and this timing is constant across all MR exams carried out in each patient.

[f]In the event of significant patient motion, a radial acquisition scheme may be used; however, this acquisition scheme can cause significant differences in ADC quantification and should be used only if EPI is not an option. Furthermore, this type of acquisition takes considerably more time.

[g]TSE is equivalent to FSE.

[h]2D FLAIR is an optional alternative to 3D FLAIR, with sequence parameters as follows per previously published recommendations[2]: 2D TSE/FSE acquisition; TE = 100-140 ms; TR = >6000 ms; TI = 2000-2500 ms (chosen based on vendor

recommendations for optimised protocol and field strength); GRAPPA ≤ 2; fat suppression; slice thickness ≤ 3 mm; orientation axial; FOV ≤ 250 mm × 250 mm; matrix ≥ 244 × 244.

[i]Flip angles for 3D TSE sequences are complicated because many utilise variable flip angle refocusing radiofrequency pulses to produce the desired image contrast. Investigators are encouraged to work with their scanner vendors to determine the ideal parameters.

[j]Investigators are encouraged to work with their scanner vendors to determine the best parallel imaging strategies, which may include SMS, CAIPI, iPAT, GRAPPA, as well as turbo or other acceleration factors. High performance MRI scanners may be capable of higher acceleration factors.

[k]Imaging times provided as an estimation only. Exact imaging times will depend upon individual scanner and hardware performance capabilities.

**Supplementary Table S2. Imaging features of PCNSL in immunocompetent patients**

| |
|---|
| • PCNSLs appear as single or multifocal, strongly and homogenously enhancing lesions |
| • Diffusion characteristics reflect hypercellularity that may also be recognised as hyperattenuation on unenhanced CT imaging |
| • Haemorrhage and necrosis are classically absent |
| • Perilesional oedema is less extensive than in malignant gliomas |
| • Perfusion changes are dominated by increased permeability but absent neovascularisation |
| • Perfusion changes lead to smaller increases of blood volume and flow, allowing differentiation of PCNSL from glioblastoma with a reported pooled sensitivity and specificity of 91% and 89%, respectively[3] |
| • Proton MR spectroscopy findings indicative of PCNSL include a characteristic increased choline peak, high mobile lipid peaks and variable decreases in *N*-acetyl-L-aspartic acid and creatine, which reflect increased tumour cell proliferation and massive phagocytosis of free fatty acids by macrophages |
| • This MR spectroscopy pattern is particularly helpful in differentiating PCNSL from non-neoplastic mimics |
| • To assess treatment response and recurrence, serial brain MRI following the recommended scan protocol and timing is recommended, while recognising the ongoing development of improved imaging-based response markers |
| • There is an unmet clinical need for better prognostication of PCNSL to exploit the potential of functional imaging combined with advanced computational approaches, such as radiomics |

CT, computed tomography; MR, magnetic resonance; MRI, magnetic resonance imaging; PCNSL, primary central nervous system lymphoma.

**Supplementary Table S3. Histopathological and molecular findings in PCNSL**

| |
|---|
| • Microscopically, neoplastic B lymphocytes display a diffuse, dense arrangement, often accompanied by perivascular positioning of lymphomatous cells at the edge of these masses |
| • An exclusive interstitial, non-mass forming growth pattern is rare |
| • Neoplastic cells are large, with regular nuclei, and very often resemble centroblasts: variably but not prominently admixed with immunoblasts |
| • Mitoses and necrosis can be evident |
| • A diagnostic IHC panel including CD20, CD3, CD10, Bcl-6, Bcl-2, MUM1 markers and Ki-67 antibodies is recommended |
| • Most PCNSLs display a 'nongerminal centre' phenotype |
| • Accompanying populations of small reactive T lymphocytes, with interstitial or perivascular distribution,[4] macrophages, microglia and other non-neoplastic cells form the microenvironment of PCNSL |
| • Classical cases do not require molecular detection of clonality of the Ig heavy and light chain loci, confirmed by direct sequencing |
| • In patients receiving corticosteroids before biopsy and thereby with fewer viable lymphomatous B cells, PCR studies for clonality may be helpful, provided the false-positive results caused by the 'pseudoclonality' phenomenon and false-negative results are considered |

Bcl, B-cell lymphoma; CD, cluster of differentiation; Ig, immunoglobulin; IHC, immunohistochemistry; MUM1, multiple myeloma 1; PCNSL, primary central nervous system lymphoma.

**Supplementary Table S4. Molecular biology features and rationale for future precision therapies**

| |
|---|
| • The constitutive activation of BCR–TLR–NF-κB signalling in PCNSL is sustained by multiple mechanisms, including:<br>o mutations in *MYD88 L265P*, *PIM1*, telomerase and *SLIT2*<br>o overexpression of the NF-κB co-activator IκB-ζ and ghrelin/growth hormone secretagogue receptor axis<br>o deletion of *PRDM1* and *TNFAIP3* genes[5-7] |
| • These defects provide the rationale for the therapeutic use of BTK inhibitors[8,9] |
| • PCNSL cells exploit the PI3K–mTOR–Akt and Ras/MAPK signalling pathways[10,11] |
| • Abnormalities in the above-mentioned pathways are not mutually exclusive, suggesting that their combined targeting could be more effective than inhibiting one pathway in isolation |
| • PD-1 is expressed by some microenvironmental players, including lymphocytes and macrophages, while PD-L1 has been reported in a subgroup of both neoplastic lymphocytes and microenvironmental components in a substantial proportion of patients with PCNSL[12] |
| • Targeting both BCR–TLR–NF-κB signalling and XPO1 may have a direct effect on neoplastic cells as well as attenuating PD-1 and SIRPα expression on microenvironmental M2-like macrophages, thus skewing these latter cells toward a proinflammatory M1-like profile[13] |

Akt, protein kinase B; BCR, B-cell receptor; BTK, Bruton tyrosine kinase; MAPK, mitogen-activated protein kinase; mTOR, mammalian target of rapamycin; NF-κB; nuclear factor-κB; PCNSL, primary central nervous system lymphoma; PD-1, programmed cell death protein 1; PD-L1, programmed death-ligand 1; PI3K, phosphoinositide 3-kinase; Ras, rat sarcoma virus; SIRPα, signal regulatory protein alpha; TLR, toll-like receptor; XPO1; exportin 1.

**Supplementary Table S5. Pretreatment assessments and staging work up in PCNSL**

| | |
|---|---|
| **Involvement of CNS compartments** | • Neurological examination<br>• Gadolinium-enhanced MRI of the brain and spine[a]<br>• CSF analyses (physical–chemical exam; conventional cytology; flow cytometry, Ig genes assessment by PCR, *MYD88* mutational status, IL-10 level)[14]<br>• Ophthalmological examination (including slit lamp fundoscopy and retinal angiography or tomography) |
| **Dissemination outside the CNS** | • FDG–PET–CT total body[b]<br>• Contrast-enhanced total-body CT scan<br>• Testicular US[c]<br>• Bone marrow biopsy and aspiration[d]<br>• Symptoms-driven specific exams |
| **Pretreatment assessment and risk definition** | • Medical history, including concomitant medication and corticosteroid use<br>• Physical examination including PS (ECOG or Karnofsky)<br>• Full blood count; liver and renal function index; serology for HIV, HCV, HBV; serum protein electrophoresis; pregnancy test<br>• Cardiac assessment (echocardiography and electrocardiogram)<br>• Pulmonary assessment: thorax CT scan and pulmonary function test (spirometry test and diffuse capability of $CO_2$)[e]<br>• Cognitive function assessment by neuropsychological tests and QoL assessment |

| | |
|---|---|
| | • Prognostic score: IELSG score (age, PS, LDH serum level, CSF protein concentration and involvement of deep areas of the CNS) and MSKCC score (age and PS) |

CNS, central nervous system; CSF, cerebral spinal fluid; CT, computed tomography; ECOG, Eastern Cooperative Oncology Group; FDG–PET, [$^{18}$F]2-fluoro-2-deoxy-D-glucose–positron emission tomography; HBV, hepatitis B virus; HCV, hepatitis C virus; HIV, human immunodeficiency virus; IELSG, International Extranodal Lymphoma Study Group; Ig, immunoglobulin; IL-10, interleukin-10; LDH, lactic dehydrogenase; MRI, magnetic resonance imaging; MSKCC, Memorial Sloan Kettering Cancer Center; PCNSL, primary central nervous system lymphoma; PET, positron emission tomography; PS, performance status; QoL, quality of life; US, ultrasound.

[a]Spinal MRI only in symptomatic cases or if CSF positive.

[b]Contrast-enhanced neck, chest, abdomen and pelvis CT scan should be carried out if PET–CT is not feasible.

[c]In the absence of testicular enlargement, US is indicated when PET is not feasible.

[d]Bone marrow biopsy and aspiration are indicated if PET is not feasible or to investigate cytopaenias.

[e]Only for patients with known lung disease; CT of the thorax should be added when PET–CT is not feasible.

**Supplementary Table S6. (Immuno)ChT combinations in randomised trials**

| **Regimen (Trial)** | **Induction treatment details** |
|---|---|
| MATRix[15] (IELSG32) | Four 21-day courses of:<br>• Rituximab 375 mg/m$^2$, days -5 and 0<br>• MTX 3.5 g/m$^2$, day 1<br>• AraC 2 g/m$^2$ every 12 hours, days 2 and 3<br>• Thiotepa 30 mg/m$^2$, day 4 |
| MBVP[16 a] (HOVON 105/ ALLG NHL24) | Two 28-day courses of:<br>• MTX 3 g/m$^2$, days 1 and 15<br>• Teniposide 100 mg/m$^2$, days 2 and 3<br>• Carmustine 100 mg/m$^2$, day 4<br>• Prednisolone 60 mg/m$^2$, days 1-5<br>• Intrathecal MTX 15 mg in case of positive CSF after cycle 1 |
| R–MT[17 b] (CALGB/ Alliance 50202) | Four 28-day courses of:<br>• MTX 8 g/m$^2$, days 1 and 15<br>• Temozolomide<br>  ○ 150 mg/m$^2$/day, days 7-11 (cycle 1)<br>  ○ 200 mg/m$^2$/day, days 7-11 (cycles 2-4)<br>• Rituximab 375 mg/m$^2$<br>  ○ Days 3, 10, 17 and 24 (cycle 1)<br>  ○ Days 3 and 10 (cycles 2-4) |
| MPV[18 c] (ANOCEF-GOELAM) | Three 28-day courses of:<br>• MTX 3.5 g/m$^2$, days 1 and 15<br>• Procarbazine 100 mg/m$^2$/day, days 1-7<br>• Vincristine 1.4 mg/m$^2$ (dose capping 2.8 mg), days 1 and 15 |
| MT[19] (ANOCEF-GOELAM) | Three 28-day courses of:<br>• MTX 3.5 g/m$^2$, days 1 and 15<br>• Temozolomide 150 mg/m$^2$/day<br>  ○ Days 1-5 (cycle 1)<br>  ○ Days 15-19 (cycles 2 and 3) |

| | |
|---|---|
| R–MBVP[20]<br><br>(ANOCEF-GOELAM) | Two 28-day courses of:<br><br>• MTX 3 g/$m^2$, days 1 and 15<br>• Etoposide 100 mg/$m^2$, day 2<br>• Carmustine 100 mg/$m^2$, day 3<br>• Prednisone 60 mg/$m^2$, days 1-5<br><br>Followed by two cycles of R–AraC (rituximab 375 mg/$m^2$ on day 1, AraC 3 g/$m^2$ on days 1 and 2) |

ALLG, Australasian Leukaemia and Lymphoma Group; ANOCEF, Association des Neuro-Oncologues d'Expression Francaise; AraC, cytarabine; CALGB, Cancer and Leukaemia Group B; ChT, chemotherapy; CSF, cerebral spinal fluid; GOELAM, Groupe Ouest Est Leucémies Aiguës Myéloblastiques; HOVON, Hemato-Oncologie voor Volwassenen Nederland; IELSG, International Extranodal Lymphoma Study Group; MATRix, high-dose methotrexate–high-dose cytarabine–rituximab–thiotepa; MBVP, methotrexate–carmustine–teniposide–methylprednisolone; MPV, methotrexate–procarbazine–vincristine; MT, methotrexate–temozolomide; MTX, methotrexate; NHL, non-Hodgkin lymphoma; R–AraC, rituxmab–cytarabine; R–MBVP, rituximab–methotrexate–carmustine–etoposide–prednisone; R–MT, rituximab–MTX–temozolomide.

[a]With or without rituximab 375 mg/$m^2$, days 0, 7, 14, 21 (cycle 1) and 0, 14 (cycle 2).

[b]With one course (cycle 5) of AraC 2 g/$m^2$ every 12 hours, days 1 and 2.

[c]A variant of this regimen with the addition of rituximab 500 mg/$m^2$ on day 1 has also been reported.[21]

**Supplementary Table S7. Conditioning thiotepa-containing combinations used in PCNSL**

| |
|---|
| Busulfan–thiotepa combination[22]<br>• Busulfan four oral daily doses of 4 mg/kg BW/day, days -8 to -5<br>• Thiotepa 5 mg/kg, days -4 and -3 |
| TBC regimen[23,24]<br>• Thiotepa 250-300 mg/m$^2$/day, days -8 and -7<br>• Busulfan 3.2-9.6 mg/kg BW/day, days -6 to -4<br>• Cyclophosphamide 2 g/m$^2$/day, days -3 and -2 |
| TBC regimen[20]<br>• Thiotepa 250 mg/m$^2$/day, days -9, -8 and -7<br>• Busulfan 3.2 mg/kg/day, days -6 and -5, and 1.6 mg/kg on day -4 (total dose = 8 mg/kg)<br>• Cyclophosphamide 60 mg/kg/day, days -3 and -2 |
| Carmustine–thiotepa regimen[25]<br>• Carmustine 400 mg/m$^2$, day -6<br>• Thiotepa 5 mg/kg BW/day, days -5 and -4 |
| Carmustine–thiotepa 10 regimen[26]<br>• Carmustine 400 mg/m$^2$, day -6<br>• Thiotepa 2 x 5 mg/kg BW/day, days -5 and -4 |

BW, body weight; PCNSL, primary central nervous system lymphoma; TBC, thiotepa–busulfan–cyclophosphamide.

**Supplementary Table S8. RT technique and efficacy in PCNSL and PVRL**

| | |
|---|---|
| Patients with brain ± ocular lymphoma | • PCNSL is multifocal in ~40% of cases based on MRI at diagnosis, but conventional MRI is known to underestimate the extent of disease[27]<br>• Focal RT results in increased relapses in areas outside the irradiated volume[28]<br>• RT volume should include the whole brain, with the cervical spinal cord and meninges at levels C1 and C2, and the posterior two-third volume of the eyes[29,30]<br>• Some guidelines suggest RT to the whole eyes, including the anterior chamber in patients with concomitant brain and intraocular disease<br>• A dose tailored to the response after induction ChT is recommended: doses between 23.4 Gy/13 fractions and 30.6 Gy/17 fractions in patients with CR and a dose of 40 Gy/20 fractions in patients with PR<br>• The potential efficacy of a boost to the tumour site has not been proven<br>• In uncontrolled studies, reducing the dose of WBRT to 23.4 Gy/13 fractions in patients who achieved CR after induction led to minimal neurotoxicity and no apparent reduction in efficacy[31] |
| Patients with PVRL | • External beam ocular RT is rarely used alone as first-line treatment in patients with bilateral ocular involvement<br>• External beam ocular RT is also used as salvage treatment for elderly patients<br>• The RT volume usually includes both eyes because PVRL is often bilateral<br>• The whole eyes should be irradiated; some experts recommend including the optic nerves in the radiation volume[30]<br>• The usual technique to irradiate both eyes is opposing lateral fields with the isocentre set at the posterior border to reduce divergence; this allows later WBRT if needed<br>• A single eye can be irradiated using modern highly conformal RT techniques, with minimal doses to surrounding structures<br>• The recommended prescribed dose is 30-36 Gy/18-20 fractions |

| | |
|---|---|
| | • Vision-threatening retinopathy has been reported in 12% of patients; the risk of this side-effect is lower with reduced RT doses, but may be higher when ocular RT is combined with systemic treatments[32-34]<br>• Other complications include cataracts and dry eyes<br>• 65%-90% of patients with PVRL treated with upfront ocular RT alone experience relapse, usually in the CNS[34-36] |

ChT, chemotherapy; CNS, central nervous system; CR, complete remission; MRI, magnetic resonance imaging; PCNSL, primary central nervous system lymphoma; PR, partial response; PVRL, primary vitreoretinal lymphoma; RT, radiotherapy; WBRT, whole-brain radiotherapy.

**Supplementary Table S9. Experimental therapies in r/r PCNSL**

| Agent and dose | Study phase | No of patients | ORR, n (%) | mPFS, months | Comments |
|---|---|---|---|---|---|
| Temsirolimus (25 mg weekly in 6 patients and 75 mg weekly in 29 patients)[37] | II | 37 PCNSL | 20/37 (54) | 2.1 | CSF concentration was very low (2 ng/ml) |
| Buparlisib (100 mg daily)[38] | II | 4 (2 PCNSL, 2 SCNCL) | 1/4 (25) | 1.3 | CSF concentration was below the $IC_{50}$ observed to induce cell death in lymphoma cells *in vitro* |
| Ibrutinib (560 mg or 840 mg daily)[9] | I/II | 20 (13 PCNSL, 7 SCNSL) | 10/13 (77) | 4.6 | Mean CSF concentration was 0.77 ng/ml (1.7 nM) and 1.95 ng/ml (4.4 nM) in patients receiving 560 mg and 840 mg, respectively. A trend to higher CSF concentrations after 1 month of therapy was observed |
| Ibrutinib (560 mg daily)[8] | II | 44 (30 PCNSL, 14 PVRL) | 32/44 (73) | 4.8 | OS was 19.2 months |
| Dose-adjusted TEDDi-R–ibrutinib[39] | I | 18 PCNSL | 15/18 (83) | 15.3 | - |
| Ibrutinib (560 mg or 840 mg daily)–MTX (3.5 g/m$^2$ every 2 | I | 15 (9 PCNSL, 6 SCNSL) | 12/15 (80) | 9.2 | Mean CSF concentration was 3.105 ng/ml (equivalent to 7.05 nM, range 0.305-9.22). These |

| | | | | | |
|---|---|---|---|---|---|
| weeks)–R (500 mg/$m^2$ every 2 weeks)[40] | | | 8/9 PCNSL 4/6 SCNSL | | concentrations are similar to those reported in patients receiving single-agent ibrutinib |
| Tirabrutinib (320 mg or 480 mg daily)[41] | I/II | 44 PCNSL | 28/44 (64) | 2.9 | The CSF : plasma concentration ratio of tirabrutinib was 13%-18%. MTD not reached at 480 mg/day. ORRs were irrespective of mutations in *CARD11*, *MYD88* and *CD79B* |
| Lenalidomide (25 mg daily on days 1-21 of a 28-day cycle)[42] | Retro | 6 PCNSL | 3/6 (50) | - | - |
| Low-dose lenalidomide (5-10 mg daily) as maintenance[43] | Retro | 10 PCNSL | - | ≥9 in 6 ≥18 in 4 | Lenalidomide was detected in ventricular CSF in 10 of 13 patients and in 65% of CSF specimens. Mean CSF concentration was 4.9 ng/ml (range 0-16.68 ng/ml). A dose-dependent increase in CSF penetration of lenalidomide was observed |
| Lenalidomide (5-10 mg daily)–R (every 6 months) as maintenance[44] | Retro | 13 elderly PCNSL (≥70 years) | 10/13 in CR (77) | NR | Three of the five patients who progressed responded to salvage therapy and resumed lenalidomide |

| | | | | | |
|---|---|---|---|---|---|
| | | | | | maintenance in second CR. Only one treatment-unrelated death recorded |
| R (375 mg/m$^2$ day 1)–lenalidomide (20 mg daily followed by lenalidomide 10 mg daily) for 12 cycles[45] | II | 45 PCNSL or PVRL | 16/45 (36) | 7.8 | OS was 17.7 months. Limited benefit of maintenance (started and completed by 18 and 5 patients, respectively). Better outcome in PVRL versus PCNSL (mPFS of 9.2 versus 3.9 months) |
| Pomalidomide (5 mg daily)–dexamethasone (40 mg weekly)[46] | I | 25 PCNSL or PVRL | 12/25 (48) | 5.3 | - |
| Nivolumab (3 mg/kg every 2 weeks)[47] | Retro | 5 (4 PCNSL, 1 PTL with CNS+) | 5/5 (100) | 3/5 13+ to 17+ | One patient developed worsening of a baseline renal insufficiency (grade 4) that did not improve with corticosteroids, prompting discontinuation after three doses and initiation of haemodialysis (biopsy showed no evidence of interstitial nephritis) |
| Nivolumab (3 mg/kg or 100 mg flat dose every 2 weeks) ± other agents[48] [a] | Retro | 6 (5 PCNSL, 1 PTL with CNS+) | 3/6 (50) | 7 | - |

| | | | | | |
|---|---|---|---|---|---|
| Nivolumab (3 mg/kg)–dendritic cell vaccine[49] | Case report | 1 PCNSL | 1/1 (100) | CR at 10+ | - |
| Low dose nivolumab (40 mg every 2 weeks)[50] | Case report | 1 PCNSL | 1/1 (100) | CR at 24+ | - |
| Nivolumab (3 mg/kg every 2 weeks) as maintenance[51] | Case report | 1 PCNSL | 1/1 (100) | CR at 24+ | - |
| Pembrolizumab (200 mg every 3 weeks) or nivolumab (240 mg monthly)–R (375 mg/m$^2$ every 3 weeks)[52] | Retro | 6 (3 PCNSL, 3 SCNSL) | 3/6 (50) | - | Five patients received pembrolizumab while one received nivolumab. All received R |
| Pembrolizumab (200 mg every 3 weeks)[53] | II | 50 PCNSL | 13/50 (26) | 2.6 | 6-month PFS of 30% and 6-month OS of 60%. Median DoR of 10 months. No toxic deaths were reported |
| Anti-CD19 CAR T-cells[54] | Retro | 9 PCNSL | 6/9 (67)<br><br>5/9 (56) CR at 3 months | 4 | Seven patients developed grade ≥1 CRS and five experienced NT with highest grade CRS and NT of 3. No treatment-related deaths |
| Tisagenlecleucel[55]<br>0.6-6e8 CAR T-cells | I/II | 12 PCNSL | 7/12 (58) | NR | No treatment-related deaths.<br>One patient experienced grade 3 ICANS. Exploratory analysis identified T |

| | | | | | |
|---|---|---|---|---|---|
| | | | | | cell, CAR T-cell and macrophage gene signatures in CSF following infusion |
| NGR-hTNF (0.8 mg/$m^2$)–R–CHOP[56] | II | 28 PCNSL | 21/28 (75) | 6 | 15 systemic SAEs were recorded in 11 patients and 9 grade 1-2 reactions to NGR-hTNF infusion. There were no treatment-related deaths. IHC analysis of tumour tissue sections have revealed the presence of CD13 on the luminal side of tumour vessels |

AraC, cytarabine; CAR, chimeric antigen receptor; CD, cluster of differentiation; CNS+, central nervous system involvement; CR complete remission; CRS, cytokine release syndrome; CSF, cerebrospinal fluid; DoR, duration of response; $IC_{50}$, half-maximal inhibitory concentration; ICANS, immune cell-associated neurotoxicity syndrome; IHC, immunohistochemistry; mPFS, median progression-free survival; MTD, maximum tolerated dose; MTX, methotrexate; NGR-hTNF, cysteine–asparagine–glycine–arginine–cysteine–glycine peptide–tumour necrosis factor alpha; NR, not reported; NT, neurotoxicity; ORR, overall response rate; OS, overall survival; PCNSL, primary central nervous system lymphoma; PFS, progression-free survival; PTL, primary testicular lymphoma; PVRL, primary vitreoretinal lymphoma; R, rituximab; R–CHOP, rituximab–cyclophosphamide–doxorubicin–vincristine–prednisone; retro, retrospective; r/r, relapsed or refractory; SAE, serious adverse event; SCNSL, secondary central nervous system lymphoma; TEDDi–R, temozolomide–etoposide–liposomal doxorubicin–dexamethasone–ibrutinib–rituximab.

[a] Rituximab, high-dose MTX–AraC, ibrutinib.

**Supplementary Table S10. Follow-up strategy and long-term treatment sequelae**

| |
|---|
| • With modern treatments, PCNSL first relapses tend to occur early, usually within the first 2 years of follow-up,[57,58] although late relapses (up to 21 years) have been anecdotally reported[59] |
| • Mandatory assessments during follow-up should include medical history, physical examination and a gadolinium-enhanced MRI scan of the brain (or contrast-enhanced CT scan if MRI is contraindicated)[a] |
| • The use of 'ideal' and 'minimal' IPCG protocol based on 3T or 1.5T MRI is recommended[1] |
| • Nevertheless, the role of surveillance MRI remains controversial as only 6%-25% of all relapses are asymptomatic and discovered during routine follow-up imaging[60] |
| • Imaging surveillance may be of benefit, however, in potential candidates for intensified salvage treatment |
| • Ophthalmological examination and CSF analysis should be carried out if relapse is suspected, especially in patients with initial involvement of these organs |
| • Cognitive function should be assessed to help clinicians make a reasoned decision regarding both treatment and eventual support |
| • As survival rates increase, the comprehensive and longitudinal care of patients with PCNSL should include special attention to cognitive decline, which has been recognised as a significant problem in these patients, and still represents a major challenge in their treatment |
| • Cognitive decline, combined with other symptoms secondary to the lymphoma itself, its treatment, or both, can compromise health-related QoL |
| • The tumour and additional full-dose WBRT can affect cognitive function and health-related QoL in a clinically relevant and negative way[61] |
| • RT-induced brain injury can be subclassified as acute or early delayed (onset time <6 months) and late delayed[62] |
| • Late delayed neurotoxicity is associated with histopathological abnormalities and is characterised clinically by progressive and irreversible cognitive impairment that can ultimately lead to dementia |
| • Notably, there are still too many biases to draw reliable conclusions on the effects of different treatment modalities in this patient population |

| |
|---|
| • Follow-up assessments with the battery of cognitive and QoL measures proposed by the IPCG[63] should be conducted on an annual basis |
| • Liquid biopsies assessing circulating tumour cells or cell-free DNA in blood or CSF have not yet been integrated into the response assessment and follow-up of patients with PCNSL outside of clinical trials |

CSF, cerebrospinal fluid; CT, computed tomography; IPCG, International PCNSL Collaborative Group; MRI, magnetic resonance imaging; PCNSL, primary central nervous system lymphoma; QoL, quality of life; RT, radiotherapy; WBRT, whole-brain radiotherapy.

[a]The use of systematic surveillance imaging during follow-up is recommended by the IPCG criteria, but it is not supported by prospective data. This strategy detects only 20%-25% of brain relapses in asymptomatic patients. Imaging surveillance is advised, however, when suitable resources are available, to prevent rapid deterioration of performance status and neurological and cognitive functions, and their negative effects on salvage treatment.

**Supplementary Table S11. Levels of evidence and grades of recommendation (adapted from the Infectious Diseases Society of America-United States Public Health Service Grading System[a])**

**Levels of evidence**

| | |
|---|---|
| I | Evidence from at least one large randomised, controlled trial of good methodological quality (low potential for bias) or meta-analyses of well- conducted randomised trials without heterogeneity |
| II | Small randomised trials or large randomised trials with a suspicion of bias (lower methodological quality) or meta-analyses of such trials or of trials with demonstrated heterogeneity |
| III | Prospective cohort studies |
| IV | Retrospective cohort studies or case-control studies |
| V | Studies without control group, case reports, expert opinions |

**Grades of Recommendation**

| | |
|---|---|
| A | Strong evidence for efficacy with a substantial clinical benefit, strongly recommended |
| B | Strong or moderate evidence for efficacy but with a limited clinical benefit, generally recommended |
| C | Insufficient evidence for efficacy or benefit does not outweigh the risk or the disadvantages (adverse events, costs, etc.), optional |
| D | Moderate evidence against efficacy or for adverse outcome, generally not recommended |
| E | Strong evidence against efficacy or for adverse outcome, never recommended |

[a] By permission of Oxford University Press on behalf of the Infectious Diseases Society of America, from Dykewicz CA. Clin Infect Dis. 2001;33(2):139-144 [adapted from: Gross PA, Barrett TL, Dellinger EP, et al. Clin Infect Dis. 1994;18(3):421].[64]

## REFERENCES


1. Barajas RF, Politi LS, Anzalone N, et al. Consensus recommendations for MRI and PET imaging of primary central nervous system lymphoma: guideline statement from the International Primary CNS Lymphoma Collaborative Group (IPCG). *Neuro Oncol.* 2021;23(7):1056-1071.
2. Kaufmann TJ, Smits M, Boxerman J, et al.. Consensus recommendations for a standardized brain tumor imaging protocol for clinical trials in brain metastases. *Neuro Oncol.* 2020;22(6):757-772
3. Suh CH, Kim HS, Jung SC, et al. MRI as a diagnostic biomarker for differentiating primary central nervous system lymphoma from glioblastoma: A systematic review and meta-analysis. *J Magn Reson Imaging.* 2019;50(2):560-572.
4. Ponzoni M, Berger F, Chassagne-Clement C, et al. Reactive perivascular T-cell infiltrate predicts survival in primary central nervous system B-cell lymphomas. *Br J Haematol.* 2007;138(3):316-323.
5. Bruno A, Alentorn A, Daniau M, et al. TERT promoter mutations in primary central nervous system lymphoma are associated with spatial distribution in the splenium. *Acta Neuropathol.* 2015;130(3):439-440.
6. Chapuy B, Roemer MG, Stewart C, et al. Targetable genetic features of primary testicular and primary central nervous system lymphomas. *Blood.* 2016;127(7):869-881.
7. Muta H, Sugita Y, Furuta T, et al. Expression of the ghrelin/growth hormone secretagogue receptor axis and its functional role in promoting tumor growth in primary central nervous system lymphomas. *Neuropathology.* 2020;40(3):232-239.
8. Soussain C, Choquet S, Blonski M, et al. Ibrutinib monotherapy for relapse or refractory primary CNS lymphoma and primary vitreoretinal lymphoma: Final analysis of the phase II 'proof-of-concept' iLOC study by the Lymphoma study association (LYSA) and the French oculo-cerebral lymphoma (LOC) network. *Eur J Cancer.* 2019;117:121-130.
9. Grommes C, Gavrilovic IT, Kaley TJ, et al. Updated results of single-agent ibrutinib in recurrent/refractory primary (PCNSL) and secondary CNS lymphoma (SCNSL). *J Clin Oncol.* 2017;35(15_suppl):7515.
10. Marosvari D, Nagy N, Kriston C, et al. Discrepancy Between Low Levels of mTOR Activity and High Levels of P-S6 in Primary Central Nervous System Lymphoma

May Be Explained by PAS Domain-Containing Serine/Threonine-Protein Kinase-Mediated Phosphorylation. *J Neuropathol Exp Neurol.* 2018;77(4):268-273.

11. Takashima Y, Hayano A, Yamanaka R. Metabolome Analysis Reveals Excessive Glycolysis via PI3K/AKT/mTOR and RAS/MAPK Signaling in Methotrexate-Resistant Primary CNS Lymphoma-Derived Cells. *Clin Cancer Res.* 2020;26(11):2754-2766.
12. Monabati A, Nematollahi P, Dehghanian A, et al. Immune Checkpoint Molecules in Primary Diffuse Large B-Cell Lymphoma of the Central Nervous System. *Basic Clin Neurosci.* 2020;11(4):491-498.
13. Jimenez I, Carabia J, Bobillo S, et al. Repolarization of tumor infiltrating macrophages and increased survival in mouse primary CNS lymphomas after XPO1 and BTK inhibition. *J Neurooncol.* 2020;149(1):13-25.
14. Ferreri AJM, Calimeri T, Lopedote P, et al. MYD88 L265P mutation and interleukin-10 detection in cerebrospinal fluid are highly specific discriminating markers in patients with primary central nervous system lymphoma: results from a prospective study. *Br J Haematol.* 2021;193(3):497-505.
15. Ferreri AJ, Cwynarski K, Pulczynski E, et al. Chemoimmunotherapy with methotrexate, cytarabine, thiotepa, and rituximab (MATRix regimen) in patients with primary CNS lymphoma: results of the first randomisation of the International Extranodal Lymphoma Study Group-32 (IELSG32) phase 2 trial. *Lancet Haematol.* 2016;3(5):e217-e227.
16. Bromberg JEC, Issa S, Bakunina K, et al. Rituximab in patients with primary CNS lymphoma (HOVON 105/ALLG NHL 24): a randomised, open-label, phase 3 intergroup study. *Lancet Oncol.* 2019;20(2):216-228.
17. Rubenstein JL, Hsi ED, Johnson JL, et al. Intensive chemotherapy and immunotherapy in patients with newly diagnosed primary CNS lymphoma: CALGB 50202 (Alliance 50202). *J Clin Oncol.* 2013;31(25):3061-3068.
18. Abrey LE, DeAngelis LM, Yahalom J. Long-term survival in primary CNS lymphoma. *J Clin Oncol.* 1998;16(3):859-863.
19. Omuro A, Chinot O, Taillandier L, et al. Methotrexate and temozolomide versus methotrexate, procarbazine, vincristine, and cytarabine for primary CNS lymphoma in an elderly population: an intergroup ANOCEF-GOELAMS randomised phase 2 trial. *Lancet Haematol.* 2015;2(6):e251-e259.

20. Houillier C, Dureau S, Taillandier L, et al. Radiotherapy or Autologous Stem-Cell Transplantation for Primary CNS Lymphoma in Patients Age 60 Years and Younger: Long-Term Results of the Randomized Phase II PRECIS Study. *J Clin Oncol.* 2022;40(32):3692-3698.
21. Shah GD, Yahalom J, Correa DD, et al. Combined immunochemotherapy with reduced whole-brain radiotherapy for newly diagnosed primary CNS lymphoma. *J Clin Oncol.* 2007;25(30):4730-4735.
22. Montemurro M, Kiefer T, Schuler F, et al. Primary central nervous system lymphoma treated with high-dose methotrexate, high-dose busulfan/thiotepa, autologous stem-cell transplantation and response-adapted whole-brain radiotherapy: results of the multicenter Ostdeutsche Studiengruppe Hamato-Onkologie OSHO-53 phase II study. *Ann Oncol.* 2007;18(4):665-671.
23. Alimohamed N, Daly A, Owen C, et al. Upfront thiotepa, busulfan, cyclophosphamide, and autologous stem cell transplantation for primary CNS lymphoma: a single centre experience. *Leuk Lymphoma.* 2012;53(5):862-867.
24. Przepiorka D, Nath R, Ippoliti C, et al. A phase I-II study of high-dose thiotepa, busulfan and cyclophosphamide as a preparative regimen for autologous transplantation for malignant lymphoma. *Leuk Lymphoma*. 1995;17(5-6):427-433.
25. Illerhaus G, Marks R, Ihorst G, et al. High-dose chemotherapy with autologous stem-cell transplantation and hyperfractionated radiotherapy as first-line treatment of primary CNS lymphoma. *J Clin Oncol.* 2006;24(24):3865-3870.
26. Illerhaus G, Muller F, Feuerhake F, et al. High-dose chemotherapy and autologous stem-cell transplantation without consolidating radiotherapy as first-line treatment for primary lymphoma of the central nervous system. *Haematologica.* 2008;93(1):147-148.
27. Lai R, Rosenblum MK, DeAngelis LM. Primary CNS lymphoma: a whole-brain disease? *Neurology.* 2002;59(10):1557-1562.
28. Shibamoto Y, Hayabuchi N, Hiratsuka J, et al. Is whole-brain irradiation necessary for primary central nervous system lymphoma? Patterns of recurrence after partial-brain irradiation. *Cancer.* 2003;97(1):128-133.
29. Ferreri AJ, Abrey LE, Blay JY, et al. Summary statement on primary central nervous system lymphomas from the Eighth International Conference on Malignant Lymphoma, Lugano, Switzerland, June 12 to 15, 2002. *J Clin Oncol.* 2003;21(12):2407-2414.

30. Yahalom J, Illidge T, Specht L, et al. Modern radiation therapy for extranodal lymphomas: field and dose guidelines from the International Lymphoma Radiation Oncology Group. *Int J Radiat Oncol Biol Phys.* 2015;92(1):11-31.
31. Morris PG, Correa DD, Yahalom J, et al. Rituximab, methotrexate, procarbazine, and vincristine followed by consolidation reduced-dose whole-brain radiotherapy and cytarabine in newly diagnosed primary CNS lymphoma: final results and long-term outcome. *J Clin Oncol.* 2013;31(31):3971-3979.
32. Kaushik M, Pulido JS, Schild SE, et al. Risk of radiation retinopathy in patients with orbital and ocular lymphoma. *Int J Radiat Oncol Biol Phys.* 2012;84(5):1145-1150.
33. Soussain C, Malaise D, Cassoux N. Primary vitreoretinal lymphoma: a diagnostic and management challenge. *Blood.* 2021;138(17):1519-1534.
34. de la Fuente MI, Alderuccio JP, Reis IM, et al. Bilateral radiation therapy followed by methotrexate-based chemotherapy for primary vitreoretinal lymphoma. *Am J Hematol.* 2019;94(4):455-460.
35. Grimm SA, Pulido JS, Jahnke K, et al. Primary intraocular lymphoma: an International Primary Central Nervous System Lymphoma Collaborative Group Report. *Ann Oncol.* 2007;18(11):1851-1855.
36. Hormigo A, Abrey L, Heinemann MH, DeAngelis LM. Ocular presentation of primary central nervous system lymphoma: diagnosis and treatment. *Br J Haematol.* 2004;126(2):202-208.
37. Korfel A, Schlegel U, Herrlinger U, et al. Phase II Trial of Temsirolimus for Relapsed/Refractory Primary CNS Lymphoma. *J Clin Oncol.* 2016;34(15):1757-1763.
38. Grommes C, Pentsova E, Nolan C, et al. Phase II study of single agent buparlisib in recurrent/refractory primary (PCNSL) and secondary CNS lymphoma (SCNSL). *Ann Oncol.* 2016;27(suppl 6):vi106.
39. Lionakis MS, Dunleavy K, Roschewski M, et al. Inhibition of B Cell Receptor Signaling by Ibrutinib in Primary CNS Lymphoma. *Cancer Cell.* 2017;31(6):833-843.
40. Grommes C, Tang SS, Wolfe J, et al. Phase 1b trial of an ibrutinib-based combination therapy in recurrent/refractory CNS lymphoma. *Blood.* 2019;133(5):436-445.
41. Narita Y, Nagane M, Mishima K, et al. Phase I/II study of tirabrutinib, a second-generation Bruton's tyrosine kinase inhibitor, in relapsed/refractory primary central nervous system lymphoma. *Neuro Oncol.* 2021;23(1):122-133.

42. Houillier C, Choquet S, Touitou V, et al. Lenalidomide monotherapy as salvage treatment for recurrent primary CNS lymphoma. *Neurology.* 2015;84(3):325-326.
43. Rubenstein JL, Geng H, Fraser EJ, et al. Phase 1 investigation of lenalidomide/rituximab plus outcomes of lenalidomide maintenance in relapsed CNS lymphoma. *Blood Adv.* 2018;2(13):1595-1607.
44. Vu K, Mannis G, Hwang J, et al. Low-dose lenalidomide maintenance after induction therapy in older patients with primary central nervous system lymphoma. *Br J Haematol.* 2019;186(1):180-183.
45. Ghesquieres H, Chevrier M, Laadhari M, et al. Lenalidomide in combination with intravenous rituximab (REVRI) in relapsed/refractory primary CNS lymphoma or primary intraocular lymphoma: a multicenter prospective 'proof of concept' phase II study of the French Oculo-Cerebral lymphoma (LOC) Network and the Lymphoma Study Association (LYSA). *Ann Oncol.* 2019;30(4):621-628.
46. Tun HW, Johnston PB, DeAngelis LM, et al. Phase 1 study of pomalidomide and dexamethasone for relapsed/refractory primary CNS or vitreoretinal lymphoma. *Blood.* 2018;132(21):2240-2248.
47. Nayak L, Iwamoto FM, LaCasce A, et al. PD-1 blockade with nivolumab in relapsed/refractory primary central nervous system and testicular lymphoma. *Blood.* 2017;129(23):3071-3073.
48. Shmidt D, Andrey G, Polushin A, et al. PB1816 Nivolumab treatment for relapsed and refractory primary central nervous system lymphoma and primary testicular lymphoma with CNS involvement. *HemaSphere.* 2019;3(S1):832.
49. Furuse M, Nonoguchi N, Omura N, et al. Immunotherapy of Nivolumab with Dendritic Cell Vaccination Is Effective against Intractable Recurrent Primary Central Nervous System Lymphoma: A Case Report. *Neurol Med Chir (Tokyo).* 2017;57(4):191-197.
50. Chan TSY, Khong PL, Au-Yeung R, et al. Low-dose nivolumab induced durable complete response in relapsed primary central nervous system diffuse large B cell lymphoma. *Ann Hematol.* 2019;98(9):2227-2230.
51. Terziev D, Hutter B, Klink B, et al. Nivolumab maintenance after salvage autologous stem cell transplantation results in long-term remission in multiple relapsed primary CNS lymphoma. *Eur J Haematol.* 2018;101(1):115-118.

52. Ambady P, Szidonya L, Firkins J, et al. Combination immunotherapy as a non-chemotherapy alternative for refractory or recurrent CNS lymphoma. *Leuk Lymphoma.* 2019;60(2):515-518.
53. Hoang-Xuan K, Houot R, Soussain C, et al. First Results of the Acsé Pembrolizumab Phase II in the Primary CNS Lymphoma (PCNSL) Cohort. *Blood.* 2020;136(suppl 1):15-16.
54. Alcantara M, Houillier C, Blonski M, et al. CAR T-cell therapy in primary central nervous system lymphoma: the clinical experience of the French LOC network. *Blood.* 2022;139(5):792-796.
55. Frigault MJ, Dietrich J, Gallagher K, et al. Safety and efficacy of tisagenlecleucel in primary CNS lymphoma: a phase 1/2 clinical trial. *Blood.* 2022;139(15):2306-2315.
56. Ferreri AJM, Calimeri T, Ponzoni M, et al. Improving the antitumor activity of R-CHOP with NGR-hTNF in primary CNS lymphoma: final results of a phase 2 trial. *Blood Adv.* 2020;4(15):3648-3658.
57. Houillier C, Soussain C, Ghesquieres H, et al. Management and outcome of primary CNS lymphoma in the modern era: An LOC network study. *Neurology.* 2020;94(10):e1027-e1039.
58. Ferreri AJM, Cwynarski K, Pulczynski E, et al. Whole-brain radiotherapy or autologous stem-cell transplantation as consolidation strategies after high-dose methotrexate-based chemoimmunotherapy in patients with primary CNS lymphoma: results of the second randomisation of the International Extranodal Lymphoma Study Group-32 phase 2 trial. *Lancet Haematol.* 2017;4(11):e510-e523.
59. Nayak L, Hedvat C, Rosenblum MK, et al. Late relapse in primary central nervous system lymphoma: clonal persistence. *Neuro Oncol.* 2011;13(5):525-529.
60. Langner-Lemercier S, Houillier C, Soussain C, et al. Primary CNS lymphoma at first relapse/progression: characteristics, management, and outcome of 256 patients from the French LOC network. *Neuro Oncol.* 2016;18(9):1297-1303.
61. van der Meulen M, Dirven L, Habets EJJ, et al. Cognitive functioning and health-related quality of life in patients with newly diagnosed primary CNS lymphoma: a systematic review. *Lancet Oncol.* 2018;19(8):e407-e418.
62. Tofilon PJ, Fike JR. The radioresponse of the central nervous system: a dynamic process. *Radiat Res.* 2000;153(4):357-370.

63. Correa DD, Maron L, Harder H, et al. Cognitive functions in primary central nervous system lymphoma: literature review and assessment guidelines. *Ann Oncol.* 2007;18(7):1145-1151.

64. Dykewicz CA. Summary of the guidelines for preventing opportunistic infections among hematopoietic stem cell transplant recipients. *Clin Infect Dis.* 2001;33(2):139-144.
